# Systematic Tuning of the Electronic Effects in Covalent Organic Frameworks for Promoting Photocatalysis

**DOI:** 10.1021/acscentsci.5c01645

**Published:** 2025-11-18

**Authors:** He Gu, Pei Chen, Yinghui Xie, Yujie Zhao, Mengjie Hao, Zhongshan Chen, Hui Yang, Geoffrey I. N. Waterhouse, Abdullah M. Al-Enizi, Ayman Nafady, Xiangke Wang, Shengqian Ma

**Affiliations:** † College of Environmental Science and Engineering, 47840 North China Electric Power University, Beijing 102206, P.R. China; ‡ School of Chemical Sciences, 1415 The University of Auckland, Auckland 1142, New Zealand; § Department of Chemistry, College of Science, 37850 King Saud University, Riyadh 11451, Saudi Arabia; ∥ Department of Chemistry, 3404 University of North Texas, Denton, Texas 76201, United States

## Abstract

The efficiency of covalent organic framework (COF)-based photocatalysts depends on their electron transfer behavior, which can be rationalized in terms of conjugation effects and induction effects. While conjugation effects have been widely explored in COF-based photocatalysts, induction effects have largely been ignored despite being important to overall photocatalytic activity. Herein, a new isoreticular series of ordered COFs was rationally designed to determine the relative importance of conjugation and induction effects in promoting photocatalytic activity. Systematic component modulation revealed the importance of a balance of conjugation and induction effects in achieving optimum catalytic performance in COF-based photocatalysts. Our study shows that (i) induction effects lead to electrons accumulating in specific positions of COFs, while (ii) p−π and π–π conjugation enables accurate electron transfer to the electron-rich active sites, thereby facilitating photogenerated electron–hole separation and transport and boosting photocatalytic activity. One of our developed COFs (COF-3S) displayed excellent photocatalytic uranium extraction performance in contaminated groundwater and seawater. These results establish that both induction and conjugation effects affect the photocatalytic activity of COFs, which is expected to provide new insight into the rational design of high-performance COF-based photocatalysts.

## Graphical abstract

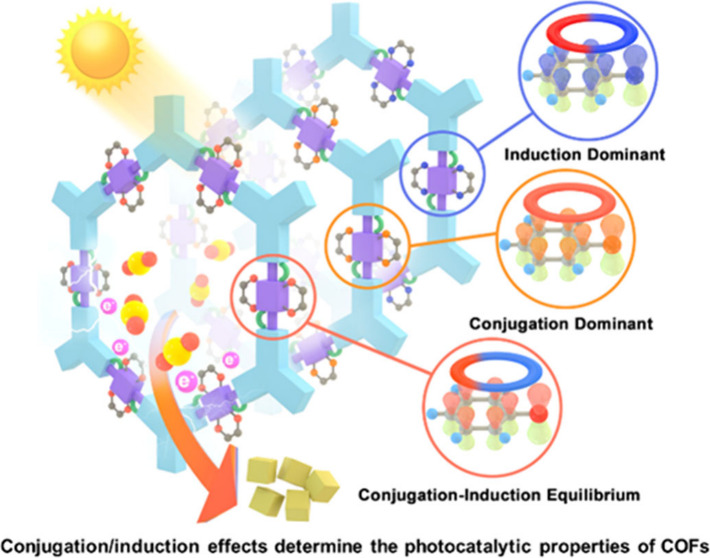

## Introduction

Covalent organic frameworks (COFs) are highly ordered porous crystalline materials composed of molecular building units connected by covalent bonds, first reported by Omar M. Yaghi and co-workers in 2005.^1^ These materials have many advantages in the targeted development of catalysts, while also allowing deep exploration of catalytic mechanisms, due to their component diversity, framework flexibility, pore geometric turnability, excellent stability, and tailorable electronic properties, among other attributes.^2–5^ Recently, the use of COFs as metal-free photocatalysts has attracted wide attention from the research community.^6–12^ Current effort is being directed toward study of the intrinsic relationship between COF structure and photocatalytic performance, research which guides the future development of high-performance COF-based photocatalysts for different applications.^13^ In recent notable contributions, researchers have explored increasing the conjugation degree in the COF frameworks as a strategy for achieving high photocatalytic activity. Accordingly, particular attention has been paid to improving the conjugation of the COF structure through linker predesign and postsynthesis approaches.^14–17^ Luo and co-workers strategically regulated the p−π conjugations of COFs by tuning the amount and position of methoxy units, significantly improving photon absorption, charge separation, and migration dynamics for improved photocatalytic hydrogen evolution performance.^18^ Jeon and co-workers reported that over- or under-conjugation causes rapid recombination of photogenerated electrons and holes. Therefore, they proposed that rationally tuning the conjugation degree of COFs could improve the photocatalytic hydrogen evolution efficiency.^19^ Subsequently, the design and synthesis of a series of planar COFs further verified that an appropriate conjugation degree is beneficial for efficient photocatalysis.^20^ Furthermore, a pore-filling technique that enhances the interlayer conjugation of COFs has been reported. This improves the photoelectric performance of COFs, which in-turn boosts photocatalytic hydrogen evolution performance.^21^ These findings identify new avenues for designing efficient COF-based photocatalysts. However, the overall photocatalytic activity of COFs is not solely determined by the degree of conjugation in their structures. In addition to the well-explored conjugation effects, there are also induction effects (which are also a type of electronic effect).^22^ Correlational studies have pointed out that tuning of electronic effects can lower the energy barrier, improve selectivity, and promote effective photocatalytic reaction processes.^23,24^ However, induction effects are often left in the too-hard basket when analyzing “structure-property” relationships in COF-based photocatalysts. Focusing only on conjugation and neglecting induction effects may cause researchers to overlook some key aspects of COFs’ photocatalytic mechanisms at the molecular level. To date, no reports have simultaneously focused on both conjugation and induction effects in relation to the photocatalytic performance of COFs. Such a study may offer valuable insights for designing improved COF photocatalysts.^25,26^

As shown in Figure 1a, conjugation effect makes the distribution of π-electrons (or p-electrons) in a conjugated system more uniform due to the increased interaction between atoms, as determined by the orbital overlap area. Moreover, π-electrons can be transferred freely through a conjugated system. Unlike the conjugation effect, induction effect pulls electrons toward more electronegative atoms (such as S, O, N, and other nonmetallic heteroatoms), increasing electron density in the heteroatom region. Based on this, it can be understood how conjugation and induction effects might influence electron transfer behavior and the formation of photocatalytic active sites, respectively, during the redox reaction processes.^27^ The relative strength of conjugation and induction effects will undoubtedly affect the photocatalytic activity of COFs, but the exact manner is still unknown. Herein, we propose a general strategy for designing a family of heteroatom-doped COFs in which the photocatalytic performance can be tuned by altering the extent of conjugation and induction effects within the COF structures (Figure 1b). Specifically, the orbital overlap area and the value of electronegativity are changed by altering the types of heteroatoms, thereby reasonably adjusting the strength of conjugation and induction effects. First, a fluorine-rich COF was synthesized through imine condensation (Figure 2a, step I). Subsequently, postsynthetic modification transformed the imine bonds into chemically stable chromenoquinoline rings through the Povarov reaction, thereby increasing π-conjugation and eliminating the directionality of the imine bonds (Figure 2a, step II). These provided a solid foundation for subsequent modification and exploration of the mechanism. Finally, various heteroatoms (e.g., N, S, O) were anchored on the framework by the replacement of the F via a substitution reaction, regulating the induction effect (Figure 2a, step III). We then conducted photocatalytic uranium removal tests from contaminated water sources to explore the relationships between the structural characteristics and photocatalytic properties of the COFs under visible light irradiation. As a result, one of our developed COFs (COF-3S) delivered a high photocatalytic uranium removal efficiency in contaminated groundwater and seawater. COF-3O, COF-3N, and their parent COFs (COF-1 and COF-2) demonstrated lower activities. Mechanism studies revealed that the balance between conjugation and induction effects determined the photocatalytic activity of our developed COFs. An appropriate conjugation degree guarantees good light absorption and sustained electron–hole pair generation during photocatalysis, while an appropriate induction effect promotes the formation of electron-rich regions, providing sufficient active sites for photocatalysis. This contribution is the first to explore the relationship between electronic effects (conjugation and induction effect) and photocatalytic performance in the COFs, guiding the rational design of high-performance photocatalysts.

**Figure 1:**
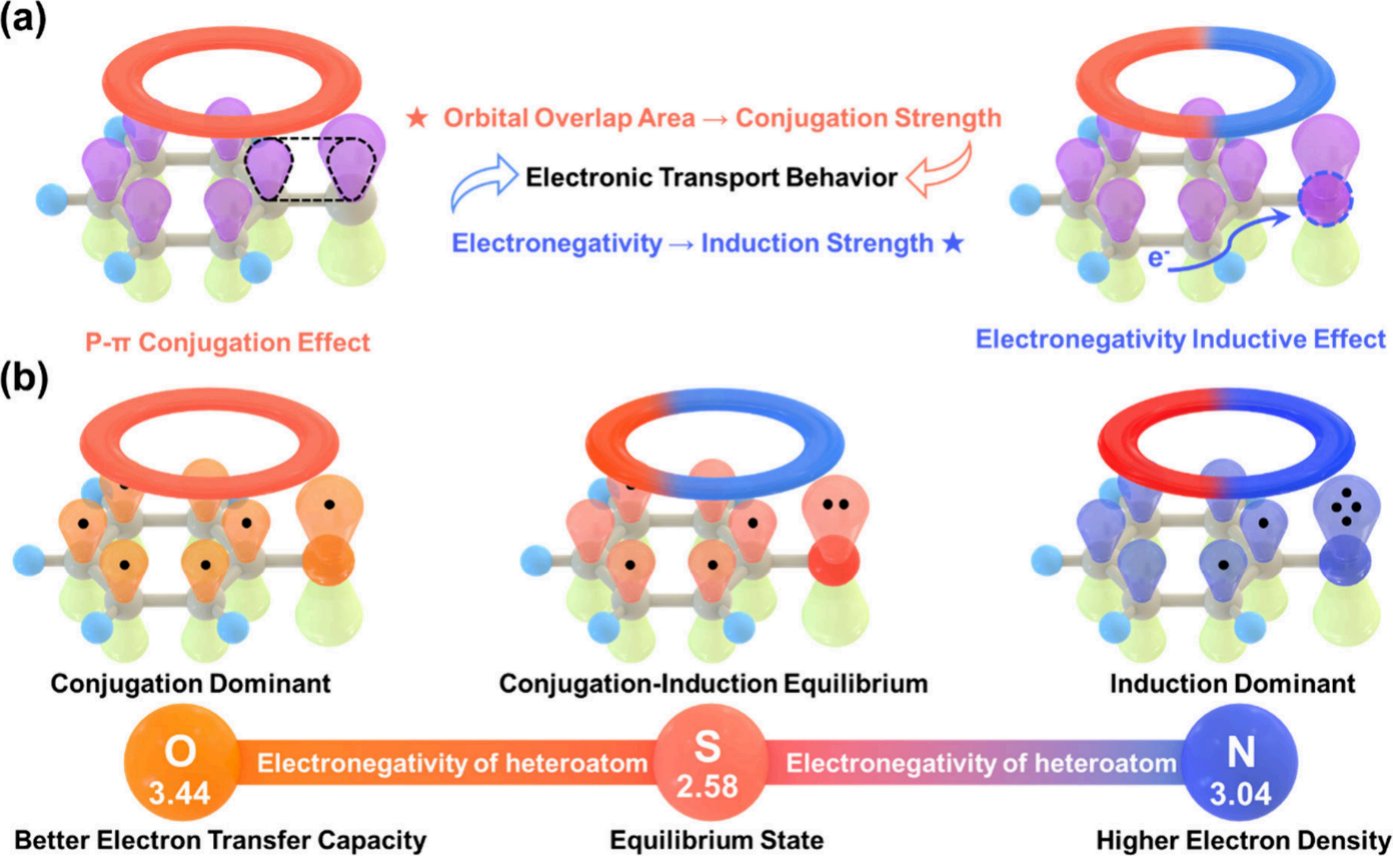
(a) Schematic illustration of conjugation and induction effects at the molecular level, highlighting the roles of conjugation and induction effects on electron transfer behavior. (b) Schematic illustration of the strategy for regulating photocatalytic performance via tuning conjugation and induction effects within the COF structure.

**Figure 2:**
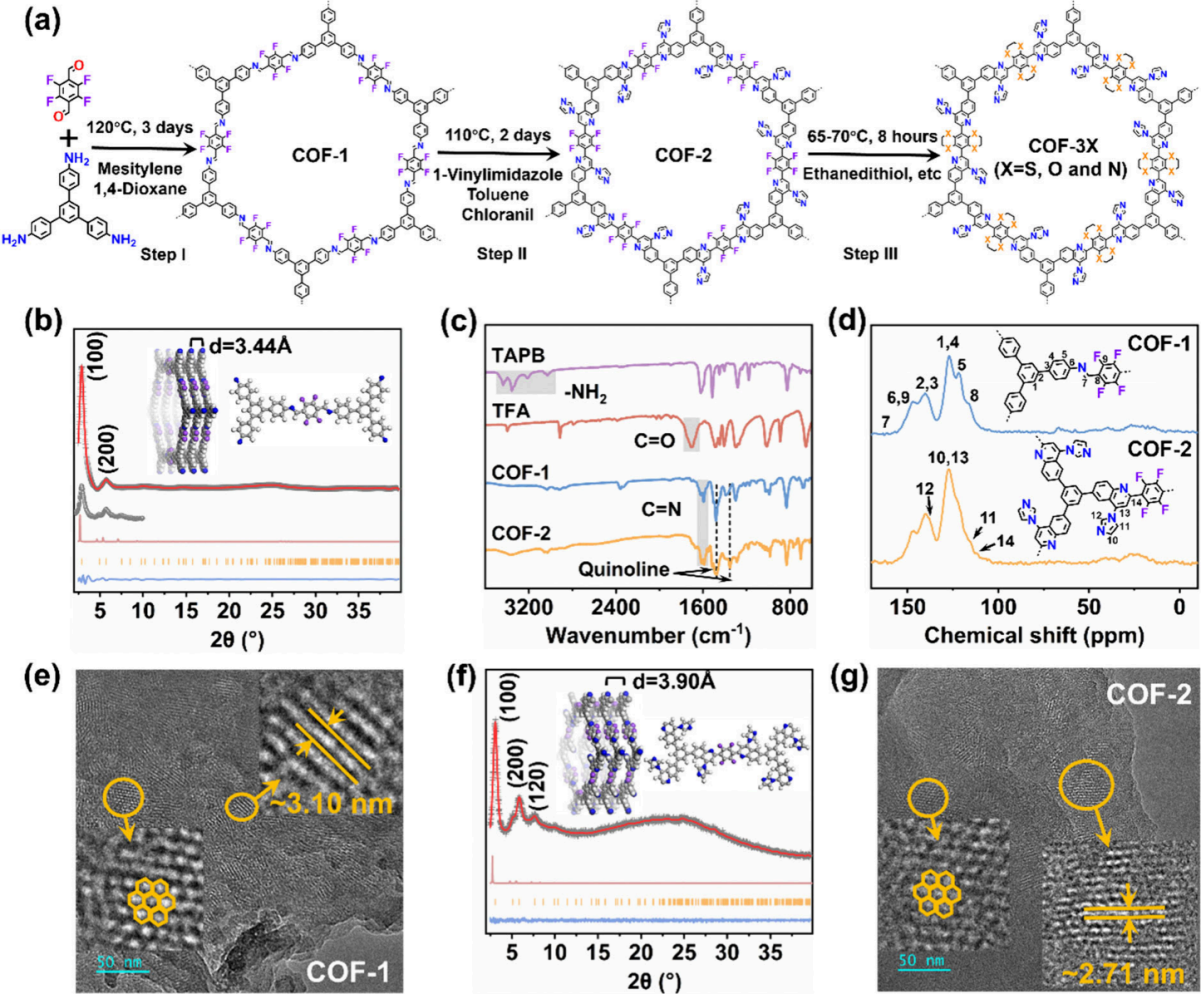
(a) Schematic illustration of the synthesis of COF-1, COF-2, COF-3S, COF-3O, and COF-3N. (b), (f) Experimental PXRD patterns of COF-1 and COF-2 with corresponding Pawley refinement (red), simulated results (light red), and Bragg positions (orange) show a good fit for the experimental data (gray) with minimal differences (blue). The inset shows the structural model and localized structural models of COF-1 and COF-2 assuming the eclipsed (AA) stacking mode. (c) FT-IR spectra of COF-1, COF-2, and linkers. (d) Solid-state ^13^C CP/MAS NMR spectra of COF-1 and COF-2. (e), (g) HRTEM image of COF-1 and COF-2 (insets highlight the honeycomb-like pores and channels).

## Results and Discussion

### Design and Synthesis of COFs

To determine the relationship between electronic effects (conjugation and induction) and photoactivity in COFs, we herein designed and synthesized a family of isoreticular COFs via the illustrations in Figure 2a. In the first step, COF-1 was synthesized via an amino-aldehyde condensation reaction of 1,3,5-tris­(4-aminophenyl)­benzene (TAPB) and 2,3,5,6-tetrafluoroterephthal-aldehyde (TFA) with 1.5 M acetic acid as the catalyst in a mixture of mesitylene and 1,4-dioxane at 120 °C for 3 days (Figure 2a, step I). The powder X-ray diffraction (PXRD) pattern for COF-1 showed a sharp diffraction peak at 2θ ∼ 2.9°, assigned to the (100) reflection, revealing the high crystallinity of COF-1 (Figure 2b). With calibration via small-angle X-ray scattering (SAXS), Pawley refinement was then applied to the experimental PXRD data of COF-1, obtaining lattice parameters of a = b = 37.99 Å, c = 3.44 Å, α = β = 90°, γ = 120° (R_wp_ = 1.31% and R_p_ = 1.59%). The structure modeling (Figure 2b, Table S1) showed COF-1 to adopt a two-dimensional (2D) parallel AA stacking mode to form honeycomb-like porous structures with a theoretical dynamic pore size of 32 Å (Figure 2b, Figure S1). The interlayer distance is about 3.44 Å due to the π–π stacking. The Fourier transform infrared spectrum (FT-IR) of COF-1 showed the appearance of two characteristic —C=N— stretching vibration signals near 1600 cm^–1^ (Figure 2c), at the expense of —NH_2_ signals at ∼3350 cm^–1^ and —C=O signals at ∼1680 cm^–1^ in the starting materials, demonstrating the successful completion of the aldimine condensation reactions.^28,29^ The solid-state ^13^C cross-polarization with magic-angle spinning (CP-MAS) NMR spectrum of COF-1 showed signals from 121 to 140 ppm ascribed to the aromatic carbons, with the peak at 147 ppm being the carbon attached to fluorine (Figure 2d).^30^ Scanning electron microscopy (SEM) and transmission electron microscopy (TEM) images showed that COF-1 possesses a microsphere morphology (Figure 2e and Figure S2). High-resolution transmission electron microscopy (HRTEM) further confirmed COF-1 possessed a honeycomb-like porous structure, which is in accordance with the structural model viewed along the c direction (Figure 2e and Figure S1). The HRTEM image of COF-1 also revealed the lattice fringes with a spacing of around 3.10 nm, which is close to the calculated one-dimensional pore diameter in the AA structure, verifying the π–π stacking (Figure 2e). The specific surface area and void fraction of COF-1 were probed by nitrogen sorption measurements at 77 K, with the adsorption and desorption curves conforming to typical type-II/type-IV sorption isotherms (Figure S3).^31,32^ The calculated Brunauer–Emmett–Teller (BET) surface area of COF-1 was 884.4 m^2^/g. These results indicated that COF-1 was successfully synthesized with a well-defined structure.

Subsequently, using boron trifluoride ether as a catalyst and tetrachlorobenzoquinone as a dehydrogenation agent, the imine bond within COF-1 was transformed into a quinoline ring structure through the Povarov reaction at 110 °C for 2 days. The resulting material was denoted as COF-2 (Figure 2a, step II). FT-IR spectrum showed signals at ∼1470 and ∼1347 cm^–1^ after the Povarov reaction, evidence for the introduction of quinoline rings (Figure 2c).^33,34^ Moreover, ^13^C CP-MAS NMR spectrum accurately also showed the successful introduction of quinoline rings in COF-2 (Figure 2d). Experimental PXRD and simulation showed that COF-2 crystallized in the P-3 space group, which was in good agreement with the Pawley refinement results (a = b = 36.97 Å, c = 3.90 Å, α = β = 90°, γ = 120°, R_wp_ = 3.07% and R_p_ = 2.44%) (Figure 2f, Table S2). Compared to COF-1, the (100) reflection of COF-2 was shifted to a higher 2θ angle (∼3.1°), suggesting a reduced pore diameter. The interlayer spacing of COF-2 was increased to 3.90 Å compared with COF-1, which was attributed to the strengthening of the π-conjugation effect between layers after the Povarov reaction (Figure 2f and Figure S4). The microsphere morphology and honeycomb-like channel (pore diameter decreased to 2.71 nm) were also retained in COF-2 (Figure 2g and Figure S5). N_2_ sorption measurements determined the BET specific surface area of COF-2 (661.1 m^2^/g) was lower than that of COF-1 (884.4 m^2^/g), which is attributed to the introduced quinoline rings occupying the pore space in COF-2 (Figure S6).

Next, we introduced different heteroatoms (e.g., S, N, O) to the COFs through a substitution reaction using ethanedithiol, ethylene glycol, or ethylenediamine as structurally similar dopants (Figure 2a, step III). By replacement of the fluorine atoms, S/O/N atoms were grafted onto the framework of COF-2, ultimately obtaining COF-3S, COF-3O, and COF-3N, respectively. PXRD and Pawley refinement analysis were employed to study the crystallinity of the newly constructed COF-3S, COF-3O, and COF-3N. The diffraction pattern of COF-3S showed intense peaks at 2θ ∼ 2.7°, 5.5°, and 7.4°, which were assigned to the (100), (200), and (120) reflections of π–π stacked 2D crystalline layers (Figure 3a,b). Pawley fitting refined unit cell parameters revealed that COF-3S crystallized in a triclinic P1 space group with unit cell parameters of a = 36.5 Å, b = 36.1 Å, c = 4.17 Å, α = β = 90°, and γ = 119.14° with R_P_ = 3.13%, R_wp_ = 3.96% (Table S3). Compared to COF-2 (3.90 Å), the interlayer distance of COF-3S was increased to 4.17 Å (Figure 3b). This might be due to variations in the space configuration and bond deflection, which resulted in a decrease in the effective overlap area of the p-orbitals.^35^ The crystalline structures of COF-3N and COF-3O were also determined using experimental PXRD data, structure modeling, and Pawley refinements (Figure 3c,d, Figures S7 and S8, Tables S4 and S5). The results showed that all these COF-3O and COF-3N exhibited similar reflections and unit cell parameters to COF-3S, indicating all possessed isostructural porous neutral frameworks. SEM images revealed that the microsphere morphology of COF-2 was retained for COF-3S, COF-3O, and COF-3N (Figure 3e and Figure S9). FT-IR spectra of COF-3S, COF-3O, and COF-3N showed that the —C=N— stretching vibrations at ∼1600 cm^–1^ and quinoline signals at ∼1470 and ∼1347 cm^–1^ were retained, indicating that the postmodification did not destroy the original structures (Figure 3f). The solid-state ^13^C NMR spectra of COF-3S, COF-3O, and COF-3N showed “fingerprint” signals for dithiane (∼40 ppm), dioxane (∼30 ppm), and piperazine (∼30 ppm) (Figure 3g), confirming the successful introduction of S, O, and N atoms, respectively.^36^ HRTEM images showed COF-3S, COF-3O, and COF-3N to consist of aggregated microspheres (Figure 3h and Figure S9). Honeycomb-like pores were observed for COF-3S, COF-3O, and COF-3N, revealing ordered alignments with high degrees of crystallinity, consistent with the porous structures determined from PXRD analysis. The N_2_ sorption isotherms of COF-3S, COF-3O, and COF-3N exhibited a type-II/type-IV adsorption profile with pore widths of ∼2.5 nm, consistent with the designed structure results (Figure 4a,b). The BET surface areas determined for COF-3S, COF-3O, and COF-3N were 559.1, 537.3, and 232.3 m^2^/g, respectively.

**Figure 3:**
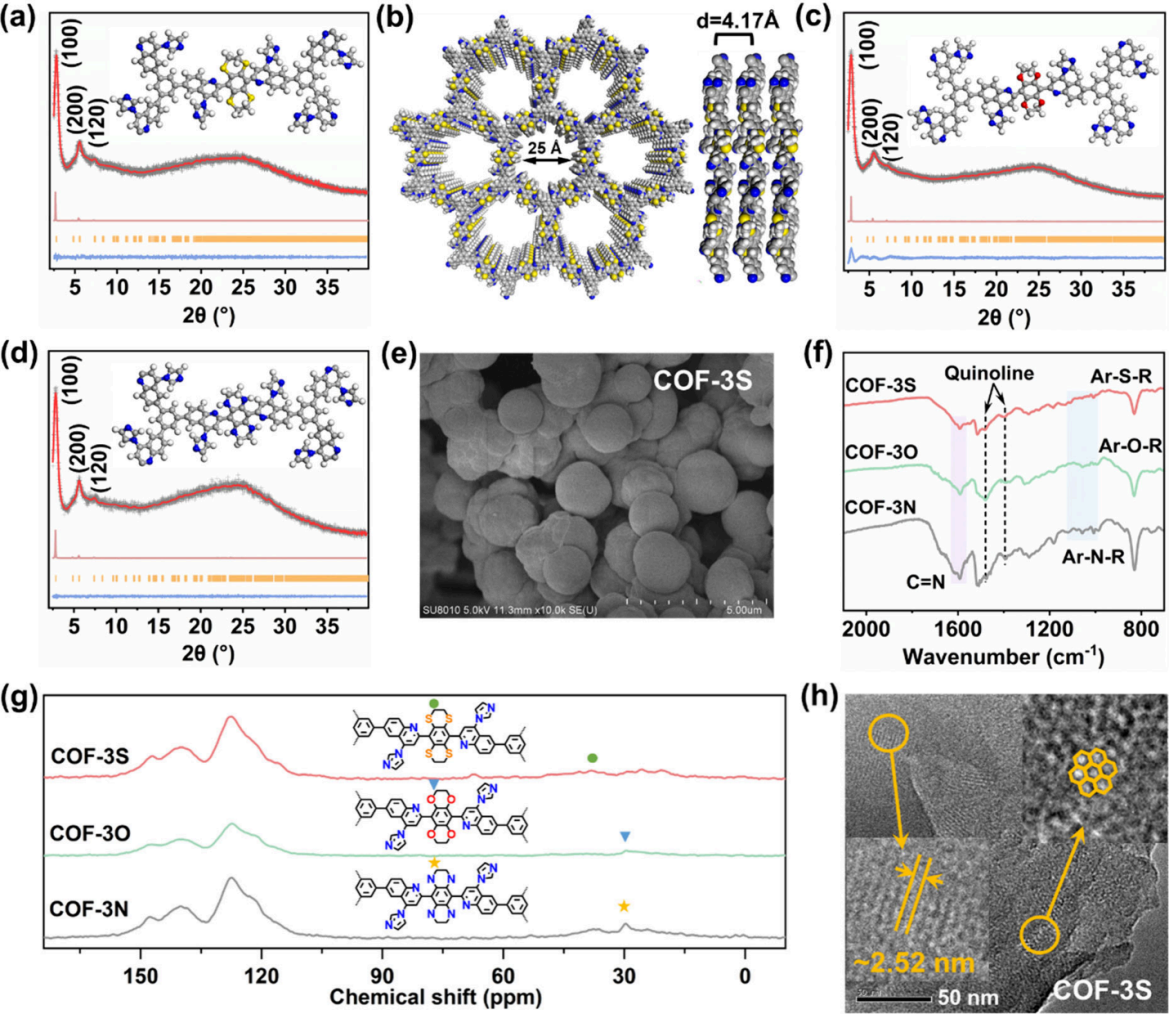
(a), (c), (d) Experimental PXRD patterns of COF-3S, COF-3O, and COF-3N with corresponding Pawley refinement (red), simulated results (light red), and Bragg positions (orange) show a good fit for the experimental data (gray) with minimal differences (blue). The inset shows the structural model and localized structural models of COF-3S, COF-3O, and COF-3N assuming the eclipsed (AA) stacking mode. (b) Top and side views of the eclipsed AA stacking crystal structure of COF-3S. The C, N, S, and H atoms are represented by gray, blue, yellow, and white spheres, respectively. (e) SEM image of COF-3S. (f) The FT-IR spectra of COF-3S, COF-3O, and COF-3N. (g) Solid-state ^13^C CP/MAS NMR spectra of COF-3S, COF-3O, and COF-3N. (h) HRTEM image of COF-3S (insets highlight the honeycomb-like pores and channels).

**Figure 4:**
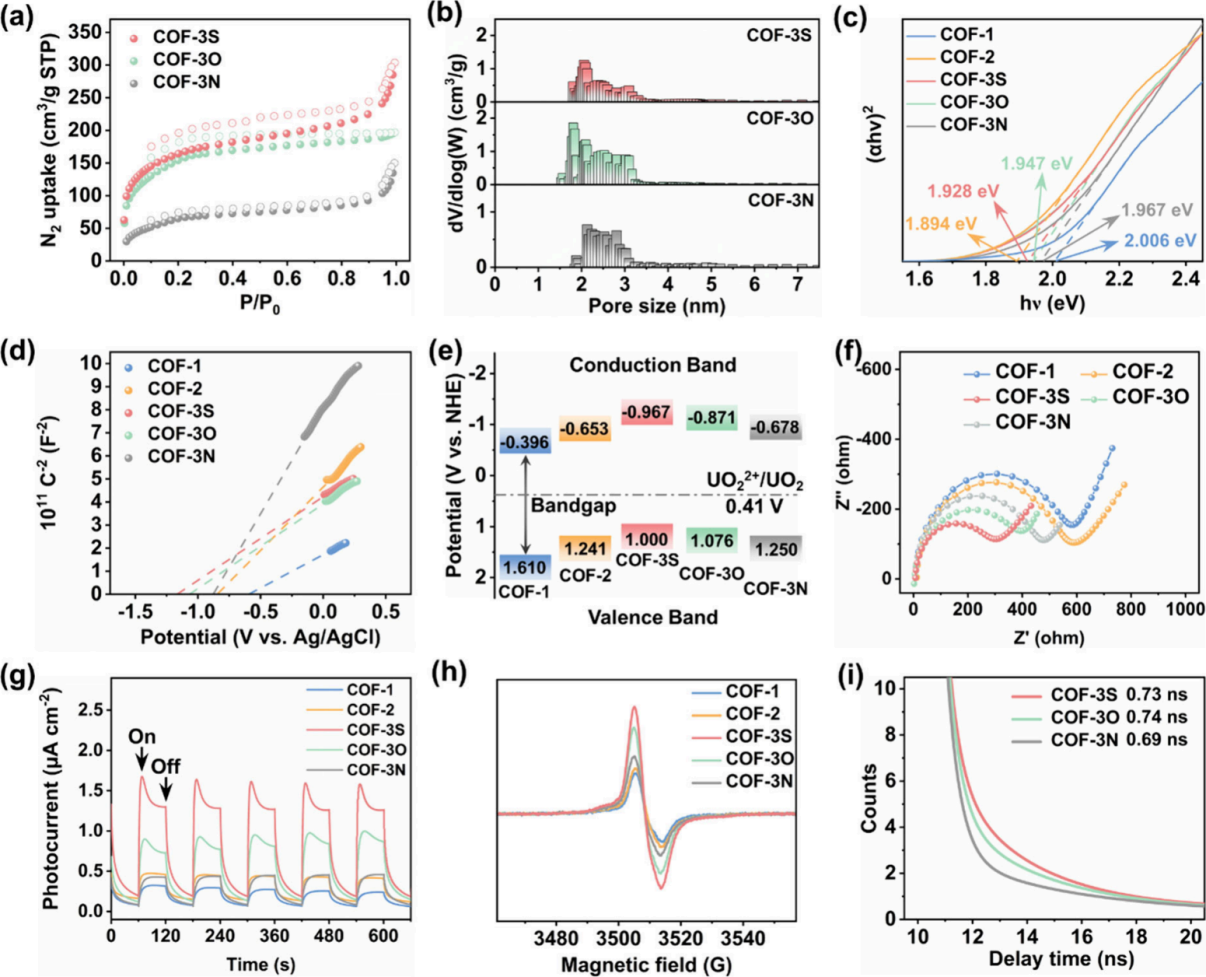
(a) N_2_ sorption isotherms measured at 77 K for COF-3S, COF-3O, and COF-3N. (b) Pore size distribution of COF-3S, COF-3O, and COF-3N determined by N_2_ physisorption at 77 K. (c) Tauc plots for COF-1, COF-2, COF-3S, COF-3O, and COF-3N. (d) Mott–Schottky plots for COF-1, COF-2, COF-3S, COF-3O, and COF-3N. (e) Band alignment for COF-1, COF-2, COF-3S, COF-3O, and COF-3N. (f) EIS spectra for COF-1, COF-2, COF-3S, COF-3O, and COF-3N. (g) Photocurrent responses for COF-1, COF-2, COF-3S, COF-3O, and COF-3N. (h) EPR conduction band e-signals for COF-1, COF-2, COF-3S, COF-3O, and COF-3N. (i) Photoluminescence decay curves for COF-3S, COF-3O, and COF-3N.

The above results demonstrate that our universal strategy for designing isoreticular COF photocatalysts with differing conjugation and induction effects was successful. Below, we focus on exploring the optical, electronic and photocatalytic properties of all COFs, with the aim of identifying structure–function relationships.

### Photoelectronic Properties of the COFs

UV/Visible diffuse reflectance spectroscopy (UV/vis DRS), electrochemical impedance spectroscopy (EIS), transient photocurrent responses, and electron paramagnetic resonance (EPR) spectroscopy were performed to systematically investigate the photophysical and electrochemical properties of COF-1, COF-2, COF-3S, COF-3N, and COF-3O. UV/vis DRS spectra showed that all COFs demonstrated similar light absorbance properties ranging from 255 to 475 nm (Figure S10). The UV–vis DRS spectra were further converted into Tauc plots to investigate the band gap of COFs. The determined band gaps for COF-1, COF-2, COF-3S, COF-3O, and COF-3N were 2.006, 1.894, 1.928, 1.947, and 1.967 eV, respectively (Figure 4c). These results confirmed their strong visible light harvesting ability. Subsequently, Mott–Schottky tests were carried out to determine the flat band potential (E_fb_) of each COF. As shown in Figure 4d, COF-3S and COF-3O exhibited negative E_fb_ of −1.17 V and −1.07 V versus an Ag/AgCl electrode, much lower those that of COF-3N (−0.877 V), COF-1 (−0.6 V), and COF-2 (−0.85 V). The positive slopes in Figure 4d indicate these COFs are n-type semiconductors.^37^ Using the E_fb_ values, the valence band potentials (E_VB_, vs NHE) were determined to be 1.610, 1.241, 1.000, 1.076, and 1.250 eV for COF-1, COF-2, COF-3S, COF-3O, and COF-3N, respectively (Figure 4e). Figure 4e compares the energy levels of the five COFs, as well as the UO_2_^2+^/UO_2_ redox couple (−0.41 V vs NHE). The data suggest that all COFs possessed suitable bandgap structures for the photocatalytic reduction of U­(VI) to U­(IV).^38,39^ Electrochemical impedance spectroscopy (EIS) was next carried out to probe the electronic conductivity of COF-1, COF-2, COF-3S, COF-3O, and COF-3N. The Nyquist plots of COF-1 possessed the largest semicircle diameter among all COFs, with semicircle diameters decreasing in the order COF-1 > COF-2 > COF-3N > COF-3O > COF-3S (Figure 4f). This indicates that the formation of the quinoline ring and the introduction of heteroatoms were beneficial in reducing the charge transfer resistance of the COFs (with COF-3S possessing the lowest charge transfer resistance). COF-3S and COF-3O demonstrated stronger photocurrent responses compared to COF-1, COF-2, and COF-3N, indicating that they offered the best charge separation efficiency (Figure 4g). Compared to COF-3N, COF-3S and COF-3O showed stronger photocurrent responses and smaller EIS Nyquist plots, implying a faster charge transfer rate and more efficient charge carrier separation ability.^40^ The presence of heteroatoms clearly facilitates the separation of photogenerated electrons and holes, thus inducing the accumulation of electrons. Analogously, the generation of photogenerated charge carriers could also be followed by electron paramagnetic resonance (EPR) spectroscopy. As shown in Figure 4h, the EPR results demonstrated that all COFs displayed signals around g = 2.000, indicating the formation of excited state electrons under visible light excitation.^41^ The trend in the EPR signal intensity for all COFs was consistent with the photocurrent response measurements, verifying the quinoline ring and heteroatoms improved the photoelectric performance. These results were also reflected in the photoluminescence (PL) lifetime data. COF-3N, COF-3O, and COF-3S exhibited a PL lifetime of 0.69, 0.74, and 0.73 ns, respectively (Figure 4i and Figure S11). The longer PL lifetimes of COF-3O and COF-3S reflect their excellent interfacial charge separation and migration capacity.^42^ By comparison, COF-3N showed a shorter PL lifetime, implying faster electron–hole pair recombination following photoexcitation (which should be detrimental to photocatalysis).

Taken together, the optoelectronic characterization of all COFs indicated that the introduction of quinoline ring and heteroatom-doped COFs (notably COF-3S and COF-3O) displayed better visible-light-harvesting capacity, more negative E_fb_ position, lower electrochemical impedance, and more efficient photocatalytic electron generation and accumulation performance compared to COF-1 and COF-2. Moreover, COF-3S showed the best balance of conjugation effects and induction effects, followed by COF-3O and COF-3N. To assess the relative merits of the COFs, in terms of their conjugation and induction attributes, a series of photocatalytic uranium extraction experiments were carried out.

### Photocatalytic Uranium Extraction and Mechanism Studies

As a strategic resource, uranium is an indispensable fuel in nuclear power applications.^43^ However, with the ongoing mining of uranium ores and the expansion of the nuclear industry, uranium contamination in groundwater has become increasingly severe, posing significant potential threats to both ecological systems and human health.^44,45^ On the flipside to remediation, demand for uranium is expected to increase in the future as a fuel for nuclear fission reactors, motivating the search for adsorbents which can selectively mine uranium from seawater. In recent years, COF photocatalysts have demonstrated remarkable compatibility and exceptional performance in addressing the dual challenges (uranium capture from wastewater and uranium mining from seawater), positioning themselves as a promising and viable solution on both counts. We thus evaluated the photocatalytic uranium extraction performance of COF-3S, COF-3N, COF-3O, and their parent frameworks COF-1 and COF-2, as photocatalysts for uranium extraction from contaminated groundwater and seawater. This aim is to delve deeper into the intrinsic relationship between the microstructure of COFs and their photocatalytic properties, thereby providing a theoretical foundation for the structural optimization of COF-based photocatalysts.

We first evaluated the photocatalytic performance of COFs in ∼30 ppm uranyl-spiked groundwater (Figure 5a). As expected, COF-3S displayed the fastest uranyl removal rates among all COFs, reaching a uranyl removal efficiency of 94.0% after 14 h. The uranyl removal efficiencies of COF-3O and COF-3N were slightly lower than that of COF-3S under similar conditions, achieving 93.6% and 90.9% (after 14 h), respectively. The extraction efficiency of COF-2 was 84.9%, higher than that of COF-1 (71.1%) under similar conditions. The photocatalytic activity of COF-1, COF-2, COF-3S, COF-3N, and COF-3O were further evaluated by conducting uranium extraction experiments from uranyl-spiked seawater (∼30 ppm), with the catalytic efficiency also following the order COF-3S (removal efficiency up to 69.1% in 4 h) > COF-3O (removal efficiency up to 39.8% in 4 h) > COF-3N > COF-2 > COF-1 (Figure 5b). These results revealed that COF-3S exhibited the highest photocatalytic activity among all of the COFs, which suggested very efficient charge carrier generation and utilization under visible light irradiation. Subsequently, COF-3S was selected as the optimal material to investigate the ability to selectively remove uranyl ions in the presence of multiple metal ions. As expected, COF-3S could achieve a removal efficiency of 61.7% for uranyl ions, which was much higher than other metal ions (3.1% for Na^+^, 1.9% for Zn^2+^, 1.5% for Mg^2+^, 1.4% for K^+^, 0.9% for Al^3+^, 0.5% for Ni^2+^, and 0.07% for Cu^2+^), indicating its excellent selectivity (Figure S12).

**Figure 5:**
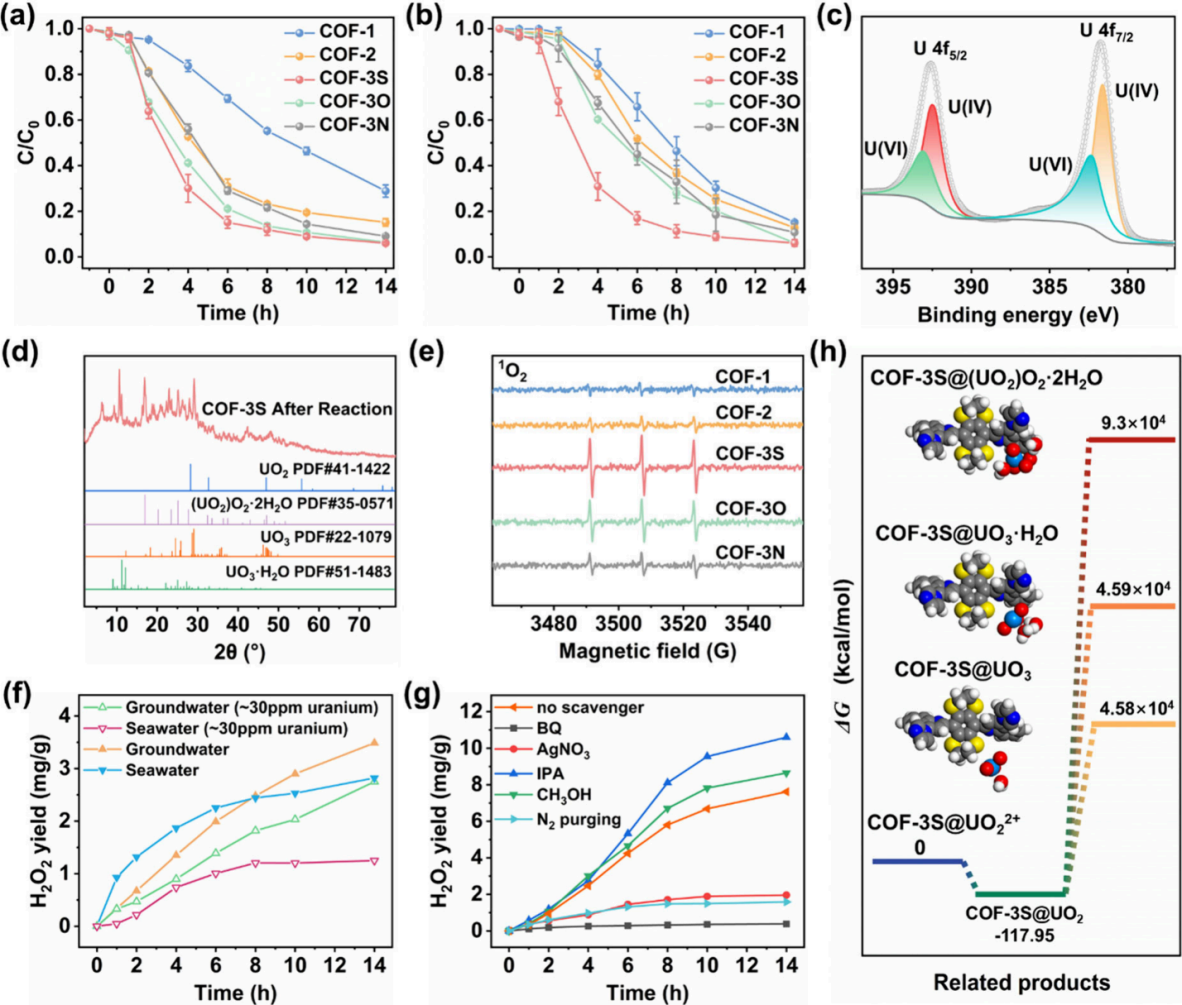
(a, b) Uranium extraction from uranyl-spiked groundwater and uranyl-spiked seawater with initial uranium concentrations of ∼30 ppm, using COF-1, COF-2, COF-3S, COF-3O, and COF-3N as photocatalysts. (c) U 4f XPS spectrum of COF-3S after photocatalysis. (d) Experimental PXRD patterns of COF-3S after reaction, containing the standard PDF cards of UO_2_, (UO_2_)­O_2_·2H_2_O, UO_3_·H_2_O, and UO_3_. (e) EPR spectra for ^1^O_2_-TEMP complexes formed under visible light irradiation of COF-1, COF-2, COF-3S, COF-3O, and COF-3N. (f) Photocatalytic H_2_O_2_ production performance of COF-3S in groundwater or seawater. (g) Quenching experiments for photocatalytic H_2_O_2_ production. (h) The relative free energy diagrams of uranyl removal on COF-3S.

To further understand the photocatalytic uranium extraction mechanism, we conducted extensive experimental studies and theoretical calculations on the COFs to verify the charge carrier separation and transition pathways under visible light irradiation. X-ray photoelectron spectroscopy (XPS) was employed to determine the surface valence state of generated uranium products after photocatalysis (Figure 5c).

The U 4f XPS spectrum after photocatalytic reaction was resolved into peaks due to U­(VI) at 382.30 and 393.03 eV in 4:3 area ratio, and peaks due to U­(IV) at 381.60 and 392.42 eV, confirming the existence of both U­(VI) and U­(IV) species on the surface of COF-3S.^46,47^ Furthermore, the PXRD pattern of COF-3S after photocatalysis in uranium-spiked groundwater comprised a mixture of (UO_2_)­O_2_·2H_2_O, UO_3_·H_2_O, UO_3_, and UO_2_ (Figure 5d). These indicated that COF-3S successfully released electrons to reduce U­(VI) to U­(IV) under the action of light (initially producing UO_2_), with some of the U­(IV) reoxidized into substances such as (UO_2_)­O_2_·2H_2_O, UO_3_·H_2_O and UO_3_ under the action of some photocatalytic reaction byproducts (such as H_2_O_2_, •OH, and ^1^O_2_) through redox pathways.^48–50^ Therefore, we conducted EPR studies to identify the radicals generated during photocatalytic processes by the COFs under visible light irradiation. As expected, ^1^O_2_, •OH, and •O_2_^–^ were trapped and detected by employing 3,4-dihydro-2,3-dimethyl-2H-pyrrole 1-oxide (DMPO), and 2,2,6,6-tetramethylpiperidine (TEMP) as trapping agents under visible light irradiation, respectively (Figure 5e and Figure S13). On the basis of these results, we next detected the H_2_O_2_ production performance of COF-3S in groundwater and seawater, which helps to understand the generation of U­(VI) species such as (UO_2_)­O_2_·2H_2_O, UO_3_·H_2_O, and UO_3_. As a result, the H_2_O_2_ production capacities in groundwater and seawater reached 3.49 and 2.82 mg/g, respectively (Figure 5f). The H_2_O_2_ production capacities were reduced to 2.75 (groundwater) and 1.25 mg/g (seawater) in the presence of uranium under similar conditions (Figure 5f). The results are explained by competition for electrons in the production of H_2_O_2_ and conversion of uranyl ions to (UO_2_)­O_2_·2H_2_O. Subsequently, we conducted a series of free radical trapping experiments using scavengers to determine the production of H_2_O_2_ pathways.^51,52^ The H_2_O_2_ production capacity was reduced to 1.58 mg/g after purging with N_2_, and similar results were observed by adding the AgNO_3_ as an electron scavenger (Figure 5g). By contrast, the H_2_O_2_ production capacity was restored after purging with O_2_. When 1,4-benzoquinone (BQ) is used for eliminating •O_2_^–^, the obtained H_2_O_2_ production capacity was 0.38 mg/g.^51,52^ The H_2_O_2_ production capacity increased to 10.60 mg/g in the presence of isopropanol (IPA), which is used to eliminate the •OH.^51,52^ These results revealed that the production of H_2_O_2_ depends on the reduction of dissolved O_2_ by photocatalytically generated electrons and confirmed that the photocatalytic oxygen reduction reaction (ORR) was a key pathway for the photocatalytic generation of H_2_O_2_ by COF-3S. Taken together, these results agreed well with the reported photocatalytic UO_2_^2+^ → UO_2_ → (UO_2_)­O_2_·2H_2_O pathway (UO_2_^2+^ + 2e^–^ = UO_2_, UO_2_ + 2H_2_O_2_ = (UO_2_)­O_2_·2H_2_O (s)).^53^

Next, we performed density functional theory (DFT) calculations to verify the adsorption and redox pathways of UO_2_^2+^ by COF-3S at the molecular level. The calculated results are shown in Figure 5h. UO_2_^2+^ binds to the active site of COF-3S to produce COF-3S@UO_2_^2+^, which serves as the starting point for the calculations. Subsequently, two electrons were transferred from COF-3S to UO_2_^2+^, generating the COF-3S@UO_2_ under visible light irradiation. The Gibbs free energy of COF-3S@UO_2_ was calculated to be −117.95 kcal/mol, which required energy consumption. This indicates that the COF-3S@UO_2_ is an unstable intermediate. Subsequently, the COF surface attached UO_2_ was oxidized to (UO_2_)_2_·2H_2_O, UO_3_·H_2_O, and UO_3_ after generation of •OH, ^1^O_2_, and •O_2_^–^ free radicals. During this step, the calculated Gibbs free energies of COF-3S@(UO_2_)_2_·2H_2_O, COF-3S@UO_3_·H_2_O, and COF-3S@UO_3_ were 9.3 × 10^4^, 4.59 × 10^4^, and 4.58 × 10^4^ kcal/mol, respectively. The COF-3S@(UO_2_)_2_·2H_2_O showed the highest Gibbs free energy for the release of COF-3S@UO_2_, explaining its identity as the primary final product. These results were consistent with the previous experimental results.

Overall, the heteroatom-doped COFs exhibited superior photocatalytic performance, mainly manifested in a more negative conduction band position, lower electron transfer resistance, and more substantial photogenerated electron generation and accumulation capacity than the pristine COFs. However, scientific issues have emerged, namely, what caused heteroatom-doped COFs to exhibit superior performance. In recent years, as catalysts, COFs have possessed donor–acceptor (D–A) structure, even donor–acceptor–acceptor (D–A–A) structure, realizing effective photogenerated charge separation and electron migration.^54^ In this work, COF-3S, COF-3O, and COF-3N also belong to this type of structure. TAPB was regarded as an electron-donating segment, with the remaining parts containing heteroatoms considered to be electron-accepting sectors. Through the Povarov reaction, the quinoline ring eliminated the electron transfer barrier of the original imine bond, enabling electrons from the donor region to transfer to the acceptor region under the electron-donating conjugation effect. Whereafter, electron-rich regions, also known as the active sites, formed in the electron-accepting sector. With a vast potential energy difference, the active sites combined with UO_2_^2+^ in the system and reduced them. According to the experimental results of the photocatalytic reaction, COF-3S exhibited the best U­(VI) removal efficiency among the heteroatom-doped COFs. Therefore, compared to the O and N atoms in COF-3O and COF-3N, it might be inferred that the S atoms in COF-3S could possess a higher charge, resulting in stronger reactivity. Therefore, materials characterization, DFT theoretical calculations, and experimental comparisons were carried out successively to obtain deeper insights into the mechanism.

### Relationship between Conjugation and Induction Effects in the COF Photocatalysts

Inhomogeneous electrostatic potential (ESP) distribution analysis is generally used for charge carrier separation and transport in photocatalysts.^55–57^ However, the ESP distribution of COF-3S was more homogeneous than those of COF-3O and COF-3N (Figures 6a, b, and c), which is opposite to our previous speculation. We further calculated the charge of each atom in the COFs via Bader charge calculations. The results are summarized in Table S6 and Figures 6a, b, and c. The extreme charges of S atoms at the reactive sites were −46.38 and −46.39 kcal/mol, respectively, lower than those of O (−47.91 and −47.60 kcal/mol) and N (−47.52 and −47.52 kcal/mol) atoms located at the same position in the COFs. These indicated that, under certain specific circumstances, local polarization of the framework in the initial state might not necessarily be the most critical factor in endowing COFs with excellent photocatalytic activity. Some other structural characteristics must have enabled COF-3S to exhibit the best photocatalytic activity, even if the S sites did not possess the maximum charge.

**Figure 6:**
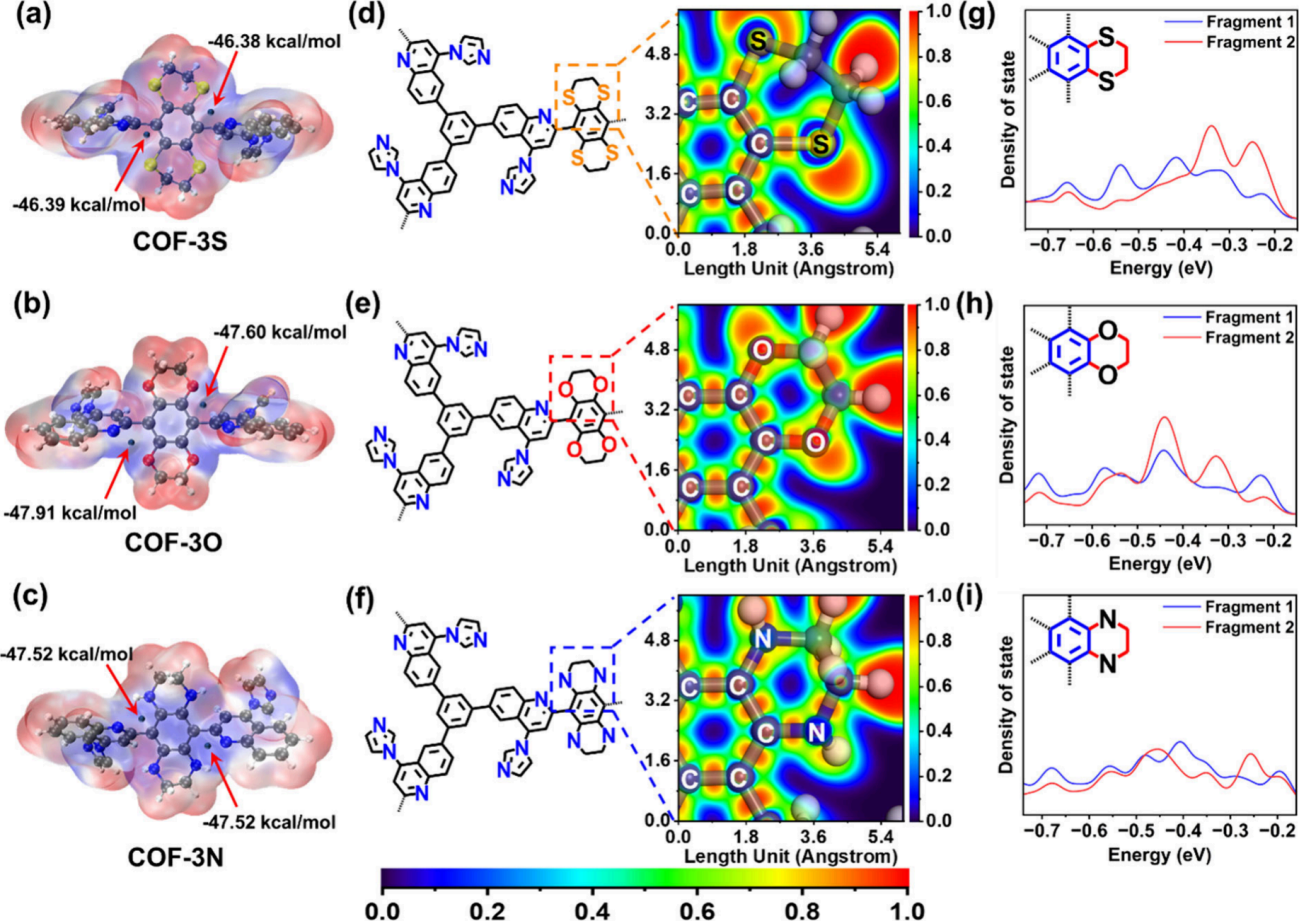
(a, b, c) The electrostatic potential diagrams of COF-3S, COF-3O, and COF-3N. (d, e, f) The localized ELF diagrams of COF-3S, COF-3O, and COF-3N. (g, h, i) The PDOS curves of COF-3S, COF-3O, and COF-3N.

Considering that the extreme charge states in the initial configuration do not directly correlate with the photocatalytic activity of the COFs, we postulated that this phenomenon might be intrinsically linked to the electron transfer feasibility during photocatalysis, which depended on the strength of conjugation and induction effects within the material. As mentioned above, under the bonding interaction forces between atoms, the entry of heteroatoms into the COF framework would cause structural distortion and transformation, thereby affecting the effective overlap area of electronic orbitals and electron clouds between atoms.^58,59^ The ultimate manifestation was the changes in the combined effects of conjugation and induction. In previous studies, conjugation and induction effects were often proposed and interpreted along with electron cloud structure diagrams (making it easier for the reader to understand). However, the electron clouds and atomic orbitals we mentioned earlier were hypothetical concepts that only represented the probability of electron distribution in a specific space. Therefore, we employed the electron localization function (ELF) to qualitatively and quantitatively verify our hypothesis via Multiwfn, which could reflect the localized and shared behavior of electrons in molecules, providing information about chemical bonds, lone pair electrons, and interatomic interactions.^60,61^ The ELF diagrams for COF-3S, COF-3N, and COF-3O are shown in Figures 6d, e, and f. In COF-3S, the maximum ELF value of lone pair electrons of the S atom was 0.95. Moreover, the ELF value between the C atoms connected to S was approximately 0.917. For COF-3O and COF-3N, the maximum ELF values of lone pair electrons of O and N atoms were 0.90 and 0.99, respectively. The ELF values between the C atoms connected to O and N atoms were approximately 0.885 and 0.924. Obviously, among the three materials, COF-3S possessed a moderate electron binding force, while the N electron binding force in COF-3N was too strong, and O was completely opposite. These results show that excessive or insufficient ELF values weaken the photocatalytic activity of COFs. A suitable electron binding force could ensure the formation of electron-rich regions while allowing electrons to transfer along conjugated P-orbitals to the electron acceptor.^62^

We further calculated the projected density of states (PDOS) to determine the conjugation degree of COF-3S, COF-3N, and COF-3O.^63–65^ The results revealed that COF-3O has the highest degree of overlap, followed by COF-3S, while COF-3N has the lowest degree of overlap (Figures 6g, h, and i). These results indicated that the N atom in COF-3N exhibited an induction effect as the dominant factor, while the O atom in COF-3O mainly demonstrated a conjugation effect. COF-3S achieved a delicate balance between conjugation effects and induction effects.

To validate the utility of the delicate balance of conjugation and induction effects for enhanced photocatalytic activity of COFs, we further designed and synthesized COF-4S, COF-4O, and COF-4N (Figure S14). The synthesis and characterization data for COF-4S, COF-4O, and COF-4N are provided in the Supporting Information (Figures S14–S20; Tables S7–S9). COF-4S, COF-4O, and COF-4N did not contain quinoline rings, thus reducing the π-conjugation. The calculated ELF values were 0.928, 0.927, and 0.929 for COF-4S, COF-4O, and COF-4N, respectively (Figure S21). These data are higher than those of the corresponding COF-3S, COF-3O, and COF-3N (0.921, 0.920, and 0.920), suggesting that the presence of a quinoline ring significantly increased π-conjugation, offsetting the partial binding force of nitrogen atoms on electrons. COF-4S, COF-4O, and COF-4N further showed much lower photocatalytic activities toward uranium removal from spiked groundwater, consistent with the above theoretical results (Figure S22).

To sum up, the results of experiments and theoretical calculations indicated that the synergistic effect of conjugation and induction effects generated by heteroatoms can significantly affect the photocatalytic activity of heteroatom-doped COFs. If the conjugation effect is excessively strong, it will result in an overly uniform charge distribution, leading to fewer active sites. An excessive induction effect can impede the transfer of electrons from other positions to the active site, causing a reduction or even cessation of the reaction rate. Hence, these two effects must be balanced, ensuring the accumulation of a certain number of electrons and the formation of active sites while allowing electrons from other positions to flow smoothly toward the active sites.

## Conclusion

In summary, we designed and synthesized a series of heteroatom-doped COFs (COF-3S, COF-3O, and COF-3N) and investigated the relationship between conjugation-induction effects that determine their photocatalytic properties at the molecular level. The experimental and theoretical results showed that although COF-3S did not have active sites with the highest charge, the more optimal balance of conjugation and induction effects in its structure afforded the best photocatalytic activity. We expect that this study will provide new ideas for the design of COF-based photocatalysts for various applications.

## Supplementary Material





## Notes

Experimental procedures, characterization data, supplemental figures, supplemental tables, and computational details (PDF)
Transparent Peer Review report available (PDF)

## Abbreviations

COF: covalent organic framework
TAPB: 1,3,5-tris­(4-aminophenyl)­benzene
TFA: 2,3,5,6-tetrafluoroterephthal-aldehyde
IPA: isopropanol
BQ: 1,4-benzoquinone
