# Supplementary material for: Systematic Tuning of the Electronic Effects in Covalent Organic Frameworks for Promoting Photocatalysis
Source: ACS Cent Sci. 2025 Nov 18;11(12):2448–59. doi: 10.1021/acscentsci.5c01645 (PMC12746150; doi:10.1021/acscentsci.5c01645)
Supplement: Supplementary file 1 [file oc5c01645_si_001.pdf]

# Supporting Information

# Systematic Tuning of the Electronic Effects in Covalent Organic Frameworks for Promoting Photocatalysis

He Gu[†], Pei Chen[†], Yinghui Xie[†], Yujie Zhao[†], Mengjie Hao[†], Zhongshan Chen[†], Hui Yang*[,†], Geoffrey I. N. Waterhouse[‡], Abdullah M. Al-Enizi[§], Ayman Nafady[§], Xiangke Wang*[,†], and Shengqian Ma*[,‖]

[†] College of Environmental Science and Engineering, North China Electric Power University, Beijing 102206, P.R. China

[‡] School of Chemical Sciences, The University of Auckland, Auckland 1142, New Zealand

[§] Department of Chemistry, College of Science, King Saud University, Riyadh 11451, Saudi Arabia

[‖]Department of Chemistry, University of North Texas, Denton, Texas 76201, United States

Hui Yang: h.yang@ncepu.edu.cn
Xiangke Wang: xkwang@ncepu.edu.cn
Shengqian Ma: shengqian.ma@unt.edu

# Chemicals and Instrumentation

### Chemicals

1,3,5-tris(4-aminophenyl)benzene (TAPB) and 2,3,5,6-tetrafluoroterephthal-aldehyde (TFA) were purchased from Jilin Chinese Academy of Sciences-Yanshen Technology Co., Ltd. Ethanol, acetone, 1,4-dioxane, acetic acid (AcOH), mesitylene, toluene, tetrahydrofuran, ethanedithiol, ethylene glycol, ethylenediamine, boron (tri)fluoride etherate, chloranil, 1-vinylimidazole, potassium carbonate, cesium carbonate, N,N-Diisopropylethylamine, N,N-Dimethylacetamide (DMAc), N,N-Dimethylformamide (DMF), sodium acetate, chloroacetic acid, arsenazo III, Nafion solution, sodium sulfate, potassium iodide, and potassium hydrogen phthalate were purchased from Shanghai Macklin Biochemical Co., Ltd (Shanghai, China). All chemicals were used without further purification. Ultrapure water was obtained from a Millipore system (18.25 MΩ·cm). The seawater was collected in Maoming, Guangdong Province, China. The underground water was collected from the Mentougou District, Beijing, China.

### Instrumentation

Powder X-ray diffraction (PXRD) patterns were collected on a Rigaku SmartLab SE X-ray diffractometer equipped with a Cu Kα source. Fourier-transform infrared spectra (FT-IR) were recorded on a SHIMADZU IRTracer-100. $^{13}C$ solid-state cross-polarization magic angle spinning nuclear magnetic resonance ($^{13}C$ CP/MAS NMR) spectra were recorded on a Bruker AVANCE III 400 WB spectrometer. Scanning electron microscopy (SEM) images were recorded on a HITACHI-SU8010 scanning electron microscope. High−resolution transmission electron microscopy (HRTEM) images were recorded on JEOL JEM−2100F and JEM-F200 transmission electron microscopes operating at an accelerating voltage of 200 kV. Brunauer-Emmett-Teller (BET) surface areas and pore size distributions were determined from $N_2$ adsorption/desorption isotherms collected at 77 K using a Micromeritics TriStar II. UV-vis spectra of the COFs were recorded in diffuse reflectance (DR) mode at room temperature on a Hitachi UH4150 spectrophotometer equipped with an integrating sphere attachment. Photoelectrochemical experiments were carried out on a CHI760 workstation. Photocurrent tests were carried out under visible light irradiation, which was generated by a 300 W xenon lamp (PerfectLight, PLS-SXE300). Electron paramagnetic resonance (EPR) spectra were obtained via a Bruker A200 spectrometer. Inductively coupled plasma mass spectrometry (ICP−MS) analyses were performed on an Agilent 7800 spectrometer. X-ray photoelectron spectroscopy (XPS) analyses were performed using a Thermo Scientific ESCALAB 250Xi spectrometer, equipped with a monochromatic Al Kα X−ray source. All binding energies were calibrated against the C 1s peak at 284.8 eV of neutral hydrocarbons. Thermogravimetric analyses (TGA) were carried out on a NETZSCH STA 2500 instrument. Samples were heated under an $N_2$ atmosphere from 25 to 800 °C at a heating rate of 10 °C/min. Photoluminescence (PL) spectra were obtained at room temperature on an Edinburgh FLS1000 spectrofluorometer operating at an excitation wavelength of 375 nm.

# Material synthesis

### Synthesis of COF-1

1,3,5-Tris(4-aminophenyl)benzene (TAPB, 21.10 mg) and 2,3,5,6-tetrafluoroterephthalaldehyde (TFA, 18.55 mg) were dissolved in a mixed solvent solution containing 1,4-dioxane/mesitylene (1 mL, 1/9 by v/v) in a 5 mL glass tube. Next, the mixture was sonicated for 10 min after which 0.1 mL acetic acid (0.1 mL, 1.5 M) was added. Afterwards, the mixture was frozen by placing the glass tube in a liquid $N_2$ bath (77 K), degassed by three freeze-pump-thaw cycles, sealed under vacuum, and then heated at 120 °C for 3 days. The solid product was collected by filtration and washed with ethanol, water, and tetrahydrofuran (each three times). Finally, the product powder was dried at 45 °C for 12 h under vacuum, yielding COF-1.

### Synthesis of COF-2

COF-1 (25 mg) was mixed with chloranil (36.9 mg), 1-Vinylimidazole (16.32 μL) and toluene (2 mL) in a 5 mL glass tube. Next, the mixture was sonicated for 10 min, after which boron trifluoride etherate (18.8 μL) was added. Subsequently, the mixture was frozen by placing the galls tube in a liquid $N_2$ bath (77 K), degassed by three freeze-pump-thaw cycles, and sealed under vacuum. The tube was then heated at 110 °C for 2 days, after which the solid product was collected by filtration and washed with ethanol, saturated sodium bicarbonate, and tetrahydrofuran (each three times). Finally, the product powder was dried at 45 °C for 12 h under vacuum, yielding COF-2.

### Synthesis of COF-3S, COF-3O, and COF-3N

COF-2 (25 mg) and N,N-diisopropylethylamine (2 mL) were stirred in N,N-dimethylacetamide (DMAc, 5 mL, purged with $N_2$ for 10 min beforehand) in a 25 mL three-neck, round-bottom flask. Ethanedithiol (20 μL) was then injected under $N_2$ protection. The mixture was stirred at 70 °C for 8 h, after which a dark brown powder was obtained. After cooling to room temperature, the resulting dark brown powder was collected by vacuum filtration and washed with ethanol, distilled water, and tetrahydrofuran (each three times). Finally, the powder was dried at 45 °C for 12 h under vacuum, yielding COF-3S. COF-3O and COF-3N were prepared using the same general protocol, replacing ethanedithiol with ethylene glycol (20 μL) and ethylenediamine (20 μL), and replacing diisopropylethylamine with potassium carbonate (49.75 mg) and cesium carbonate (117.29 mg), respectively.

**Table S1**. Fractional atomic coordinates for the AA-stacking unit cell of COF-1

| space group P6/M (C6H-1), a = b = 37.99 Å, c = 3.44 Å, α = β= 90°, γ = 120° Pawley Refinement $R_{wp}$ = 1.31%, $R_p$ = 1.59% | | | |
|---|---|---|---|
| Atom | x (Å) | y (Å) | z (Å) |
| C1 | 0.47254 | 0.01435 | 0 |
| C2 | 0.45813 | 9.73E-01 | 0 |
| C3 | 0.48552 | 9.58E-01 | 0 |
| C4 | 0.52862 | 8.64E-02 | 0 |
| F5 | 0.58266 | 5.32E-02 | 0 |
| F6 | 0.55476 | 9.72E-01 | 0 |
| C7 | 0.31061 | 6.86E-01 | 0 |
| C8 | 0.28988 | 6.43E-01 | 0 |
| C9 | 0.37477 | 7.56E-01 | 0 |
| C10 | 0.41779 | 7.81E-01 | 0 |
| C11 | 0.4373 | 8.23E-01 | 0 |
| C12 | 0.41468 | 8.43E-01 | 0 |
| C13 | 0.37245 | 8.20E-01 | 0 |
| C14 | 0.35274 | 7.78E-01 | 0 |
| N15 | 0.43287 | 8.87E-01 | 0 |
| H16 | 0.50604 | 9.57E-02 | 0 |
| H17 | 0.2933 | 7.01E-01 | 0 |
| H18 | 0.43775 | 7.68E-01 | 0 |
| H19 | 0.47009 | 8.40E-01 | 0 |
| H20 | 0.3545 | 8.35E-01 | 0 |
| H21 | 0.32013 | 7.63E-01 | 0 |

**Table S2**. Fractional atomic coordinates for the AA-stacking unit cell of COF-2

| space group P-3, a = b = 36.97 Å; c = 3.90 Å, α = β = 90° and γ = 120° Pawley Refinement $R_{wp}$ = 3.07%, $R_p$ = 2.44% | | | |
|---|---|---|---|
| Atom | x (Å) | y (Å) | z (Å) |
| C1 | -0.01819 | -5.40E-01 | -0.16283 |
| C2 | 0.02567 | -5.14E-01 | -0.16038 |
| C3 | 0.04363 | -4.75E-01 | 0.00094 |
| F4 | -0.03705 | -5.77E-01 | -0.34418 |
| F5 | 0.05062 | -5.25E-01 | -0.34044 |
| C6 | 0.08909 | -4.48E-01 | 0.0082 |
| C7 | 0.3716 | -1.79E-01 | 0.33689 |
| C8 | 0.35377 | -2.22E-01 | 0.385 |
| C9 | 0.37362 | -2.43E-01 | 0.25807 |
| C10 | 0.41273 | -2.20E-01 | 0.09517 |
| C11 | 0.43195 | -1.76E-01 | 0.06241 |
| C12 | 0.41015 | -1.56E-01 | 0.17489 |
| C13 | 0.35341 | -2.90E-01 | 0.28767 |
| C14 | 0.30967 | -3.15E-01 | 0.29169 |
| N15 | 0.11217 | -4.62E-01 | 0.13652 |
| C16 | 0.14939 | -3.78E-01 | -0.07334 |
| C17 | 0.10811 | -4.04E-01 | -0.0983 |
| N18 | 0.16513 | -3.34E-01 | -0.13388 |
| C19 | 0.19822 | -3.07E-01 | -0.32419 |
| N20 | 0.20346 | -2.68E-01 | -0.32787 |
| C21 | 0.17193 | -2.71E-01 | -0.13422 |
| C22 | 0.14809 | -3.11E-01 | -0.01761 |
| H23 | 0.35616 | -1.63E-01 | 0.43284 |
| H24 | 0.32476 | -2.38E-01 | 0.52622 |
| H25 | 0.42798 | -2.36E-01 | -0.00657 |
| H26 | 0.29087 | -3.00E-01 | 0.29035 |
| H27 | 0.08774 | -3.93E-01 | -0.18749 |
| H28 | 0.21803 | -3.15E-01 | -0.46584 |
| H29 | 0.16667 | -0.24532 | -0.08065 |
| H30 | 0.12076 | -0.32317 | 0.1458 |

**Table S3**. Fractional atomic coordinates for the AA-stacking unit cell of COF-3S

| space group P1, a = 36.50 Å, b = 36.11 Å; c = 4.17 Å, α = β = 90° and γ = 119.14° Pawley Refinement $R_{wp}$ = 3.96%, $R_p$ = 3.13% | | | |
|---|---|---|---|
| Atom | x (Å) | y (Å) | z (Å) |
| C1 | 0.52598 | 0.52329 | 0.42786 |
| C2 | 0.4975 | 0.53845 | 0.38009 |
| C3 | 0.45322 | 0.50927 | 0.39805 |
| C4 | 0.43888 | 0.46845 | 0.53894 |
| C5 | 0.46902 | 0.45575 | 0.63068 |
| C6 | 0.51275 | 0.48276 | 0.56226 |
| S7 | 0.57618 | 0.54955 | 1.21472 |
| S8 | 0.51955 | 0.59577 | 0.27548 |
| S9 | 0.38109 | 0.43609 | 0.60654 |
| S10 | 0.45394 | 0.40661 | 0.85624 |
| C11 | 0.42271 | 0.52441 | 0.31338 |
| C12 | 0.15567 | 0.5613 | 0.37805 |
| C13 | 0.19874 | 0.58574 | 0.43454 |
| C14 | 0.22276 | 0.62388 | 0.2635 |
| C15 | 0.20146 | 0.63815 | 0.05877 |
| C16 | 0.15799 | 0.61463 | 0.00854 |
| C17 | 0.13588 | 0.5751 | 0.16162 |
| C18 | 0.26985 | 0.64679 | 0.26367 |
| C19 | 0.292 | 0.62461 | 0.31416 |
| C20 | 0.33485 | 0.64341 | 0.23788 |
| C21 | 0.3554 | 0.68433 | 0.10949 |
| C22 | 0.33593 | 0.70921 | 0.0955 |
| C23 | 0.293 | 0.69003 | 0.17535 |
| C24 | 0.36067 | 0.75541 | 1.01208 |
| C25 | 0.35923 | 0.62118 | 0.28617 |
| C26 | 0.39562 | 0.77164 | 0.79767 |
| C27 | 0.41897 | 0.81574 | 0.72971 |
| C28 | 0.40509 | 0.84205 | 0.8706 |
| C29 | 0.37213 | 0.82692 | 0.08613 |
| C30 | 0.35042 | 0.78417 | 0.16099 |
| C31 | 0.34229 | 0.57802 | 0.21795 |
| C32 | 0.36754 | 0.55867 | 0.22266 |
| C33 | 0.40858 | 0.5821 | 0.3489 |
| C34 | 0.4266 | 0.62489 | 0.42156 |
| C35 | 0.4037 | 0.64542 | 0.38358 |
| N36 | 0.43324 | 0.56236 | 0.40361 |
| C37 | 0.51324 | 0.97873 | 0.45659 |
| C38 | 0.52938 | 0.02182 | 0.41631 |
| C39 | 0.50347 | 0.03992 | 0.49401 |
| C40 | 0.46138 | 0.01404 | 0.59859 |
| C41 | 0.44321 | 0.97015 | 0.59672 |
| C42 | 0.46964 | 0.95166 | 0.52782 |
| S43 | 0.54815 | 0.9555 | 0.492 |
| S44 | 0.58152 | 0.05595 | 0.23195 |
| S45 | 0.43555 | 0.04238 | 0.77635 |
| S46 | 0.38609 | 0.93529 | 0.67655 |
| C47 | 0.45251 | 0.90436 | 0.57543 |
| N48 | 0.42459 | 0.88584 | 0.79556 |
| C49 | 0.523 | 0.08696 | 0.52598 |
| C50 | 0.53862 | 0.36803 | 0.34889 |
| C51 | 0.56247 | 0.34862 | 0.28675 |
| C52 | 0.60582 | 0.36839 | 0.3584 |
| C53 | 0.62408 | 0.41056 | 0.49338 |
| C54 | 0.59946 | 0.43104 | 0.54659 |
| C55 | 0.55644 | 0.40826 | 0.4783 |
| C56 | 0.63065 | 0.34496 | 0.28479 |
| C57 | 0.61166 | 0.30381 | 0.13905 |
| C58 | 0.63405 | 0.28146 | 1.08797 |
| C59 | 0.67706 | 0.30249 | 0.15284 |
| C60 | 0.69776 | 0.34304 | 0.29168 |

| | | | |
|---|---|---|---|
| C61 | 0.67368 | 0.36323 | 0.36156 |
| C62 | 0.74502 | 0.36623 | 0.3225 |
| C63 | 0.61352 | 0.23589 | 0.97184 |
| C64 | 0.76999 | 0.35062 | 0.17578 |
| C65 | 0.81087 | 0.37885 | 0.07629 |
| C66 | 0.83007 | 0.42095 | 0.18517 |
| C67 | 0.80884 | 0.4341 | 0.39254 |
| C68 | 0.76659 | 0.40716 | 0.46007 |
| C69 | 0.5774 | 0.21834 | 0.7713 |
| C70 | 0.55724 | 0.17469 | 0.68646 |
| C71 | 0.57513 | 0.14979 | 0.78502 |
| C72 | 0.61159 | 0.16694 | 0.96513 |
| C73 | 0.63025 | 0.2091 | 1.06526 |
| N74 | 0.55596 | 0.10606 | 0.7054 |
| C75 | 0.89311 | 0.44029 | -0.10919 |
| N76 | 0.87216 | 0.45019 | 0.09205 |
| C77 | 0.35318 | 0.51405 | 0.11052 |
| C78 | 0.3803 | 0.49907 | 0.15633 |
| C79 | 0.45564 | 0.83736 | 0.49926 |
| C80 | 0.46715 | 0.87749 | 0.40657 |
| C81 | 0.51717 | 0.15253 | 0.50059 |
| C82 | 0.50325 | 0.11208 | 0.40792 |
| C83 | 0.83507 | 0.36587 | 0.86065 |
| C84 | 0.87362 | 0.39559 | 0.77332 |
| N85 | 0.31087 | 0.4858 | -0.01835 |
| C86 | 0.28415 | 0.49661 | -0.16862 |
| N87 | 0.24448 | 0.46408 | 0.80809 |
| C88 | 0.24712 | 0.43094 | 0.93169 |
| C89 | 0.28721 | 0.44402 | 0.04132 |
| N90 | 0.48155 | 0.81967 | 0.38438 |
| N91 | 0.48939 | 0.17019 | 0.46546 |
| C92 | 0.46999 | 0.78018 | 0.27711 |
| N93 | 0.50278 | 0.77615 | 0.16852 |
| C94 | 0.53637 | 0.81458 | 0.22069 |
| C95 | 0.52353 | 0.84122 | 0.35095 |
| C96 | 0.49581 | 0.20445 | 0.29441 |
| N97 | 0.46187 | 0.21015 | 0.29168 |
| C98 | 0.43333 | 0.17812 | 0.47355 |
| C99 | 0.4499 | 0.15319 | 0.57732 |
| N100 | 0.81857 | 0.32245 | 0.74922 |
| C101 | 0.77803 | 0.29514 | 0.67729 |
| N102 | 0.77174 | 0.25617 | 0.5929 |
| C103 | 0.8107 | 0.26043 | 0.60507 |
| C104 | 0.83952 | 0.30119 | 0.69545 |
| C105 | 0.96658 | 0.45574 | -0.04213 |
| C106 | 0.00987 | 0.48155 | -0.08044 |
| C107 | 0.02587 | 0.52614 | -0.14932 |
| C108 | -0.00259 | 0.54232 | -0.20582 |
| C109 | 0.95418 | 0.51307 | 0.7652 |
| C110 | 0.93882 | 0.47008 | -0.14386 |
| C111 | 0.07197 | 0.55633 | -0.11871 |
| S112 | 0.91775 | 0.5277 | 0.57384 |
| S113 | 0.01768 | 0.60078 | -0.18261 |
| S114 | 0.94383 | 0.40632 | 0.19039 |
| S115 | 0.04538 | 0.45762 | 0.94083 |
| N116 | 0.09244 | 0.54798 | 0.09693 |
| C117 | 0.09493 | 0.5974 | 0.70275 |
| C118 | 0.13417 | 0.62718 | 0.77996 |
| N119 | 0.14947 | 0.67016 | 0.66097 |
| C120 | 0.18777 | 0.69791 | 0.54271 |
| N121 | 0.19145 | 0.73575 | 0.46297 |
| C122 | 0.15378 | 0.73168 | 0.53927 |
| C123 | 0.12794 | 0.69157 | 0.65907 |
| C124 | 0.54358 | 0.46622 | 0.58969 |

| | | | |
|---|---|---|---|
| N125 | 0.53042 | 0.42737 | 0.50398 |
| C126 | 0.58957 | 0.4927 | 0.6636 |
| C127 | 0.61646 | 0.47693 | 0.64467 |
| N128 | 0.6609 | 0.50522 | 0.69807 |
| C129 | 0.68602 | 0.49778 | 0.89663 |
| N130 | 0.72722 | 0.5265 | 0.85935 |
| C131 | 0.7271 | 0.55328 | 0.63122 |
| C132 | 0.68649 | 0.54081 | 0.53579 |
| C133 | 0.93443 | 0.57903 | 0.7507 |
| C134 | 0.97764 | 0.60981 | 0.62931 |
| C135 | 0.97043 | 0.38148 | 0.00058 |
| C136 | 0.01472 | 0.40424 | 0.11689 |
| C137 | 0.59929 | 0.60601 | 0.31683 |
| C138 | 0.56885 | 0.61548 | 0.49878 |
| C139 | 0.37403 | 0.38958 | -0.17534 |
| C140 | 0.40174 | 0.37277 | 0.70011 |
| C141 | 0.38009 | 1.00713 | 0.75284 |
| C142 | 0.37262 | 0.968 | 0.93085 |
| C143 | 0.59692 | 0.99075 | 0.29013 |
| C144 | 0.61262 | 0.03426 | 0.40734 |
| H145 | 0.13638 | 0.53124 | 0.49988 |
| H146 | 0.21275 | 0.57424 | 0.61034 |
| H147 | 0.21943 | 0.66659 | -0.07062 |
| H148 | 0.276 | 0.5928 | 0.40832 |
| H149 | 0.38713 | 0.69693 | 0.02502 |
| H150 | 0.27783 | 0.7091 | 0.16048 |
| H151 | 0.40313 | 0.74976 | 0.6792 |
| H152 | 0.36459 | 0.84945 | 0.2031 |
| H153 | 0.32636 | 0.77395 | 0.34516 |
| H154 | 0.30934 | 0.5601 | 0.17248 |
| H155 | 0.45919 | 0.64277 | 0.49598 |
| H156 | 0.41868 | 0.67907 | 0.42881 |
| H157 | 0.50594 | 0.3525 | 0.27907 |
| H158 | 0.54574 | 0.31838 | 0.16952 |
| H159 | 0.65728 | 0.42861 | 0.54112 |
| H160 | 0.5795 | 0.2884 | 0.06756 |
| H161 | 0.69446 | 0.28695 | 0.08568 |
| H162 | 0.6887 | 0.39354 | 0.47456 |
| H163 | 0.75984 | 0.31654 | 0.1482 |
| H164 | 0.82489 | 0.46564 | 0.49642 |
| H165 | 0.75156 | 0.41979 | 0.61271 |
| H166 | 0.5656 | 0.23867 | 0.67865 |
| H167 | 0.6247 | 0.14636 | 1.02711 |
| H168 | 0.65762 | 0.22002 | 1.21922 |
| H169 | 0.36914 | 0.46688 | 0.09767 |
| H170 | 0.48828 | 0.8899 | 0.20676 |
| H171 | 0.4738 | 0.09596 | 0.28487 |
| H172 | 0.89257 | 0.38541 | 0.64869 |
| H173 | 0.29296 | 0.52737 | -0.26701 |
| H174 | 0.22141 | 0.39895 | 0.94058 |
| H175 | 0.2974 | 0.42448 | 0.16943 |
| H176 | 0.43827 | 0.7552 | 0.26128 |
| H177 | 0.5684 | 0.82291 | 0.16819 |
| H178 | 0.54355 | 0.87407 | 0.41726 |
| H179 | 0.52397 | 0.22424 | 0.16365 |
| H180 | 0.40219 | 0.1729 | 0.52475 |
| H181 | 0.43408 | 0.12447 | 0.71934 |
| H182 | 0.75295 | 0.30467 | 0.69059 |
| H183 | 0.81801 | 0.23537 | 0.55508 |
| H184 | 0.87308 | 0.31431 | 0.72275 |
| H185 | 0.0796 | 0.60453 | 0.512 |
| H186 | 0.21305 | 0.69159 | 0.50301 |
| H187 | 0.14575 | 0.75656 | 0.50868 |
| H188 | 0.09617 | 0.67918 | 0.74675 |

| | | | |
|---|---|---|---|
| H189 | 0.60212 | 0.52592 | 0.71656 |
| H190 | 0.67476 | 0.47136 | 1.06168 |
| H191 | 0.75492 | 0.57973 | 0.53209 |
| H192 | 0.67689 | 0.55531 | 0.34678 |
| H193 | 0.93334 | 0.57667 | 1.016 |
| H194 | 0.9121 | 0.58989 | 0.67093 |
| H195 | 0.98402 | 0.64262 | 0.68465 |
| H196 | 0.9799 | 0.60782 | 0.36363 |
| H197 | 0.96937 | 0.3838 | -0.26219 |
| H198 | 0.95469 | 0.34727 | 0.07205 |
| H199 | 0.02978 | 0.38482 | 0.05729 |
| H200 | 0.01397 | 0.40605 | 0.38124 |
| H201 | 0.61066 | 0.62501 | 0.09161 |
| H202 | 0.62772 | 0.61468 | 0.46615 |
| H203 | 0.58336 | 0.6503 | 0.53938 |
| H204 | 0.56284 | 0.59962 | 0.73757 |
| H205 | 0.34062 | 0.36492 | -0.19668 |
| H206 | 0.38137 | 0.39752 | 0.08203 |
| H207 | 0.40223 | 0.37154 | 0.43508 |
| H208 | 0.38994 | 0.33996 | 0.79646 |
| H209 | 0.36838 | 1.00066 | 0.50263 |
| H210 | 0.36363 | 1.02245 | 0.87509 |
| H211 | 0.33892 | 0.95031 | 0.99787 |
| H212 | 0.3921 | 0.97743 | 1.15373 |
| H213 | 0.59316 | 0.98829 | 0.02575 |
| H214 | 0.62066 | 0.98084 | 0.35005 |
| H215 | 0.6114 | 0.03495 | 0.67413 |
| H216 | 0.64597 | 0.05452 | 0.33175 |

**Table S4**. Fractional atomic coordinates for the AA-stacking unit cell of COF-3O

| space group P1, a = 39.40 Å, b = 40.25 Å, c = 3.99 Å, α = β = 90°, γ = 120°, Pawley Refinement $R_p$ = 2.95%, $R_{wp}$ = 3.75% | | | |
|---|---|---|---|
| Atom | x (Å) | y (Å) | z (Å) |
| C1 | 0.54626 | 0.50655 | 0.03309 |
| C2 | 0.52217 | 0.52476 | -0.00119 |
| C3 | 0.48055 | 0.50391 | 1.00992 |
| C4 | 0.46428 | 0.46338 | 1.05038 |
| C5 | 0.48833 | 0.44512 | 1.08277 |
| C6 | 0.52998 | 0.46593 | 0.07188 |
| O7 | 0.58747 | 0.52925 | 0.01758 |
| O8 | 0.54046 | 0.56437 | -0.05981 |
| O9 | 0.4231 | 0.44088 | 1.07184 |
| O10 | 0.46992 | 0.4055 | 1.13976 |
| C11 | 0.45408 | 0.52372 | 0.97702 |
| C12 | 0.18973 | 0.54363 | 0.23387 |
| C13 | 0.23152 | 0.56735 | 0.21248 |
| C14 | 0.2505 | 0.60449 | 0.06351 |
| C15 | 0.22344 | 0.61584 | 0.93861 |
| C16 | 0.1806 | 0.59133 | 0.95262 |
| C17 | 0.16363 | 0.55477 | 0.1019 |
| C18 | 0.2974 | 0.63021 | 0.0479 |
| C19 | 0.32222 | 0.61309 | 0.04452 |
| C20 | 0.36465 | 0.63465 | 0.03819 |
| C21 | 0.38222 | 0.67622 | 0.03671 |
| C22 | 0.3602 | 0.69619 | 0.04233 |
| C23 | 0.31777 | 0.67175 | 1.04512 |
| C24 | 0.38092 | 0.74217 | 1.05824 |
| C25 | 0.39059 | 0.61404 | 0.04033 |
| C26 | 0.42288 | 0.77106 | -0.05304 |
| C27 | 0.44011 | 0.81275 | -0.02008 |
| C28 | 0.41705 | 0.82804 | 1.10059 |
| C29 | 0.37823 | 0.80175 | 1.20129 |
| C30 | 0.36106 | 0.76086 | 1.18446 |
| C31 | 0.37692 | 0.57495 | 0.93783 |
| C32 | 0.40129 | 0.55679 | 0.94092 |
| C33 | 0.44024 | 0.5774 | 0.05687 |
| C34 | 0.454 | 0.61556 | 0.16176 |
| C35 | 0.43023 | 0.63331 | 0.15265 |
| N36 | 0.46515 | 0.55923 | 0.07543 |
| C37 | 0.52653 | 0.96799 | 0.14711 |
| C38 | 0.54408 | 0.0091 | 0.12842 |
| C39 | 0.52223 | 0.02733 | 1.04324 |
| C40 | 0.48136 | 0.00066 | 0.98575 |
| C41 | 0.46484 | 0.95976 | 0.97338 |
| C42 | 0.48678 | 0.94183 | 0.05555 |
| O43 | 0.54863 | 0.95197 | 0.2742 |
| O44 | 0.58453 | 0.03295 | 0.18428 |
| O45 | 0.45579 | 0.01547 | 0.96108 |
| O46 | 0.42635 | 0.93591 | 0.85655 |
| C47 | 0.46946 | 0.89657 | 0.03368 |
| N48 | 0.43311 | 0.87027 | 1.12015 |
| C49 | 0.54119 | 0.07318 | 1.01075 |
| C50 | 0.55594 | 0.35357 | 0.92466 |
| C51 | 0.57967 | 0.33573 | 0.9339 |
| C52 | 0.61935 | 0.35497 | 1.04509 |
| C53 | 0.63324 | 0.39425 | 0.1425 |
| C54 | 0.60892 | 0.41249 | 0.1398 |
| C55 | 0.56986 | 0.39187 | 1.02701 |
| C56 | 0.64513 | 0.33413 | 1.0529 |
| C57 | 0.62752 | 0.29256 | 1.05764 |
| C58 | 0.64955 | 0.27252 | 0.06428 |

| | | | |
|---|---|---|---|
| C59 | 0.69199 | 0.29691 | 0.06703 |
| C60 | 0.71236 | 0.33844 | 0.06326 |
| C61 | 0.68753 | 0.35556 | 1.05503 |
| C62 | 0.75922 | 0.3644 | 0.08052 |
| C63 | 0.62883 | 0.2265 | 0.07899 |
| C64 | 0.78696 | 0.35304 | -0.03058 |
| C65 | 0.82971 | 0.37757 | 0.00217 |
| C66 | 0.8457 | 0.41532 | 1.12591 |
| C67 | 0.81905 | 0.42673 | 1.23558 |
| C68 | 0.77746 | 0.40215 | 1.21784 |
| C69 | 0.59007 | 0.19984 | 0.95506 |
| C70 | 0.5725 | 0.15783 | 0.96698 |
| C71 | 0.59319 | 0.14138 | 0.11315 |
| C72 | 0.6307 | 0.16706 | 0.24379 |
| C73 | 0.64797 | 0.20797 | 0.22531 |
| N74 | 0.57581 | 0.09891 | 0.13575 |
| C75 | 0.91531 | 0.43242 | 0.04323 |
| N76 | 0.88881 | 0.44176 | 1.13908 |
| C77 | 0.38822 | 0.51616 | 0.82882 |
| C78 | 0.41352 | 0.50224 | 0.83448 |
| C79 | 0.48199 | 0.84256 | -0.11015 |
| C80 | 0.49447 | 0.88095 | -0.08734 |
| C81 | 0.53245 | 0.12882 | 0.83196 |
| C82 | 0.52014 | 0.09047 | 0.83443 |
| C83 | 0.85973 | 0.36591 | -0.08768 |
| C84 | 0.89904 | 0.39212 | -0.07608 |
| N85 | 0.34828 | 0.49011 | 0.72784 |
| C86 | 0.32517 | 0.49677 | 0.52267 |
| N87 | 0.2894 | 0.46553 | 0.48087 |
| C88 | 0.29052 | 0.43808 | 0.66513 |
| C89 | 0.32668 | 0.45291 | 0.81437 |
| N90 | 0.51048 | 0.83157 | -0.20202 |
| N91 | 0.50442 | 0.13978 | 0.72705 |
| C92 | 0.51489 | 0.80318 | -0.06732 |
| N93 | 0.54753 | 0.80363 | -0.17617 |
| C94 | 0.56444 | 0.83381 | -0.38632 |
| C95 | 0.54204 | 0.8512 | -0.40124 |
| C96 | 0.50951 | 0.16878 | 0.52496 |
| N97 | 0.47634 | 0.17029 | 0.48312 |
| C98 | 0.44923 | 0.14068 | 0.66498 |
| C99 | 0.4662 | 0.12173 | 0.81248 |
| N100 | 0.84795 | 0.32637 | -0.15655 |
| C101 | 0.81879 | 0.29495 | -0.00588 |
| N102 | 0.81807 | 0.26234 | -0.09671 |
| C103 | 0.8483 | 0.27386 | -0.31111 |
| C104 | 0.86695 | 0.31309 | -0.34582 |
| C105 | 0.98778 | 0.44746 | 0.1505 |
| C106 | 0.02838 | 0.47162 | 0.12708 |
| C107 | 0.04735 | 0.51115 | 1.0368 |
| C108 | 0.02047 | 0.52495 | 0.97957 |
| C109 | 0.98003 | 0.49993 | 0.97384 |
| C110 | 0.96147 | 0.4606 | 0.05982 |
| C111 | 0.09416 | 0.53796 | 1.00009 |
| O112 | 0.95581 | 0.51412 | 0.86083 |
| O113 | 0.03611 | 0.56479 | 0.94958 |
| O114 | 0.97086 | 0.40992 | 0.28135 |
| O115 | 0.05223 | 0.45527 | 0.18298 |
| N116 | 0.12025 | 0.52965 | 0.12637 |
| C117 | 0.11204 | 0.57574 | 0.82025 |
| C118 | 0.15123 | 0.60178 | 0.8174 |
| N119 | 0.16286 | 0.64027 | 0.70989 |
| C120 | 0.19237 | 0.66374 | 0.50489 |
| N121 | 0.19435 | 0.6979 | 0.45977 |
| C122 | 0.16451 | 0.69547 | 0.64308 |

| | | | |
|---|---|---|---|
| C123 | 0.14497 | 0.66014 | 0.79517 |
| C124 | 0.55636 | 0.44591 | 0.10392 |
| N125 | 0.54505 | 0.41022 | 1.00848 |
| C126 | 0.59707 | 0.46734 | 0.24381 |
| C127 | 0.62222 | 0.45321 | 0.24988 |
| N128 | 0.66212 | 0.47903 | 0.35342 |
| C129 | 0.68497 | 0.472 | 0.55855 |
| N130 | 0.72063 | 0.50319 | 0.60624 |
| C131 | 0.7197 | 0.53098 | 0.42592 |
| C132 | 0.68379 | 0.5164 | 0.27272 |
| C133 | 0.97232 | 0.55482 | 0.87386 |
| C134 | 0.0128 | 0.5759 | 0.75758 |
| C135 | 0.99503 | 0.39283 | 0.26168 |
| C136 | 0.03461 | 0.42086 | 0.37817 |
| C137 | 0.60487 | 0.5701 | -0.00998 |
| C138 | 0.57765 | 0.58516 | 0.09939 |
| C139 | 0.40549 | 0.39993 | 1.08787 |
| C140 | 0.43282 | 0.38496 | 0.9784 |
| C141 | 0.42102 | 0.99322 | 0.77095 |
| C142 | 0.40072 | 0.95128 | 0.88354 |
| C143 | 0.59024 | 0.9764 | 0.26034 |
| C144 | 0.60251 | 0.01621 | 0.37938 |
| H145 | 0.17735 | 0.51607 | 0.35796 |
| H146 | 0.24911 | 0.55657 | 0.32452 |
| H147 | 0.23612 | 0.6441 | 0.83293 |
| H148 | 0.30815 | 0.58219 | 0.04743 |
| H149 | 0.41374 | 0.69361 | 0.0316 |
| H150 | 0.29999 | 0.6852 | 1.04492 |
| H151 | 0.43939 | 0.75953 | -0.17068 |
| H152 | 0.36083 | 0.81344 | 1.30016 |
| H153 | 0.33152 | 0.74377 | 1.27875 |
| H154 | 0.34695 | 0.55867 | 0.85761 |
| H155 | 0.48377 | 0.63183 | 0.2539 |
| H156 | 0.44326 | 0.66247 | 0.2429 |
| H157 | 0.52608 | 0.33724 | 0.8346 |
| H158 | 0.56662 | 0.30654 | 0.8442 |
| H159 | 0.6634 | 0.41074 | 0.21701 |
| H160 | 0.59597 | 0.2753 | 1.06575 |
| H161 | 0.70972 | 0.28339 | 0.07381 |
| H162 | 0.70158 | 0.38639 | 1.03979 |
| H163 | 0.77522 | 0.3255 | -0.15136 |
| H164 | 0.83085 | 0.45515 | 1.34089 |
| H165 | 0.75947 | 0.41319 | 1.31669 |
| H166 | 0.57369 | 0.21214 | 0.85101 |
| H167 | 0.64679 | 0.15505 | 0.36411 |
| H168 | 0.67658 | 0.2252 | 0.33708 |
| H169 | 0.40373 | 0.47426 | 0.7278 |
| H170 | 0.52435 | 0.90165 | -0.15095 |
| H171 | 0.4934 | 0.07191 | 0.70281 |
| H172 | 0.91997 | 0.38329 | -0.141 |
| H173 | 0.33403 | 0.52354 | 0.39712 |
| H174 | 0.26644 | 0.40898 | 0.68815 |
| H175 | 0.33618 | 0.43761 | 0.97752 |
| H176 | 0.49616 | 0.78374 | 0.12558 |
| H177 | 0.59158 | 0.84269 | -0.51946 |
| H178 | 0.54866 | 0.87645 | -0.54709 |
| H179 | 0.5366 | 0.18832 | 0.40237 |
| H180 | 0.41895 | 0.13344 | 0.68903 |
| H181 | 0.45175 | 0.09691 | 0.97535 |
| H182 | 0.79962 | 0.2957 | 0.18467 |
| H183 | 0.8563 | 0.25476 | -0.43366 |
| H184 | 0.89248 | 0.3304 | -0.49994 |
| H185 | 0.09329 | 0.5834 | 0.68591 |
| H186 | 0.21202 | 0.65634 | 0.38293 |

| | | | |
|---|---|---|---|
| H187 | 0.15749 | 0.71815 | 0.66462 |
| H188 | 0.12001 | 0.65009 | 0.96164 |
| H189 | 0.60703 | 0.4953 | 0.35086 |
| H190 | 0.67601 | 0.44496 | 0.67956 |
| H191 | 0.74372 | 0.56018 | 0.40882 |
| H192 | 0.67449 | 0.53199 | 0.11121 |
| H193 | 0.95472 | 0.56285 | 0.71122 |
| H194 | 0.97004 | 0.56362 | 1.13136 |
| H195 | 0.02512 | 0.60736 | 0.78061 |
| H196 | 1.01637 | 0.56932 | 0.49133 |
| H197 | 0.9821 | 0.36672 | 0.42075 |
| H198 | 0.99525 | 0.38337 | 0.00199 |
| H199 | 0.05291 | 0.40706 | 0.36359 |
| H200 | 0.03368 | 0.4284 | 0.64121 |
| H201 | 0.6317 | 0.58387 | 0.14704 |
| H202 | 0.61463 | 0.57857 | -0.27121 |
| H203 | 0.5733 | 0.58254 | 0.37465 |
| H204 | 0.59111 | 0.61602 | 0.03487 |
| H205 | 0.39486 | 0.39045 | 1.34566 |
| H206 | 0.37919 | 0.38692 | 0.92453 |
| H207 | 0.41924 | 0.35401 | 1.03992 |
| H208 | 0.43738 | 0.388 | 0.70349 |
| H209 | 0.40074 | 0.00386 | 0.79946 |
| H210 | 0.42892 | 0.99498 | 0.50363 |
| H211 | 0.3904 | 0.94857 | 1.14497 |
| H212 | 0.37445 | 0.93386 | 0.72623 |
| H213 | 0.60418 | 0.96404 | 0.42051 |
| H214 | 0.60074 | 0.97718 | 0.00202 |
| H215 | 0.59374 | 0.01509 | 0.64227 |
| H216 | 0.63484 | 0.03471 | 0.36574 |

**Table S5**. Fractional atomic coordinates for the AA-stacking unit cell of COF-3N

| space group P1, a = 37.08 Å, b = 36.75 Å; c = 4.17 Å, α =β = 90°, γ = 119.96° Pawley Refinement $R_p$ = 2.98%, $R_{wp}$ = 3.78% | | | |
|---|---|---|---|
| Atom | x (Å) | y (Å) | z (Å) |
| C1 | 0.14425 | 0.53474 | 0.5478 |
| C2 | 0.09205 | 0.55059 | 0.52079 |
| C3 | -0.05342 | 0.52508 | 0.48157 |
| C4 | -0.14333 | 0.48356 | 0.46998 |
| C5 | -0.0913 | 0.46773 | 0.49703 |
| C6 | 0.05367 | 0.49327 | 0.53626 |
| N7 | 0.29975 | 0.56159 | 0.58676 |
| N8 | 0.20334 | 0.59247 | 0.53325 |
| N9 | -0.29743 | 0.4566 | 0.43095 |
| N10 | -0.20207 | 0.42584 | 0.48457 |
| C11 | -0.10371 | 0.54151 | 0.45267 |
| C12 | -0.45581 | 0.5632 | 0.18685 |
| C13 | -0.49334 | 0.58739 | 0.22984 |
| C14 | -0.34465 | 0.62687 | 0.25229 |
| C15 | -0.16482 | 0.64194 | 0.23033 |
| C16 | -0.13779 | 0.61864 | 0.18683 |
| C17 | -0.27834 | 0.57852 | 0.1654 |
| C18 | -0.37068 | 0.65204 | 0.29853 |
| C19 | -0.38178 | 0.63203 | 0.32234 |
| C20 | -0.37974 | 0.65526 | 0.36604 |
| C21 | -0.37492 | 0.6993 | 0.38586 |
| C22 | -0.36611 | 0.72014 | 0.36281 |
| C23 | -0.36763 | 0.69615 | 0.3192 |
| C24 | -0.33883 | 0.76677 | 0.38408 |
| C25 | -0.36318 | 0.63367 | 0.39099 |
| C26 | -0.16232 | 0.78891 | 0.42122 |
| C27 | -0.1345 | 0.83284 | 0.44155 |
| C28 | -0.27206 | 0.85445 | 0.42301 |
| C29 | -0.4463 | 0.8328 | 0.38615 |
| C30 | -0.48376 | 0.78941 | 0.36711 |
| C31 | -0.19403 | 0.59564 | 0.37589 |
| C32 | -0.17027 | 0.57538 | 0.39936 |
| C33 | -0.30454 | 0.59478 | 0.43949 |
| C34 | -0.47398 | 0.63235 | 0.45469 |
| C35 | -0.50771 | 0.65134 | 0.43044 |
| N36 | -0.26134 | 0.57632 | 0.46523 |
| C37 | -0.14398 | 0.99558 | 0.53414 |
| C38 | -0.11454 | 1.03901 | 0.54966 |
| C39 | 0.0288 | 1.05427 | 0.52523 |
| C40 | 0.13767 | 1.0253 | 0.48477 |
| C41 | 0.1058 | 0.9819 | 0.46896 |
| C42 | -0.03392 | 0.96664 | 0.49358 |
| N43 | -0.29403 | 0.98147 | 0.55998 |
| N44 | -0.24301 | 1.06731 | 0.59021 |
| N45 | 0.29198 | 1.03898 | 0.45902 |
| N46 | 0.22947 | 0.95353 | 0.42792 |
| C47 | -0.07161 | 0.92072 | 0.47625 |
| N48 | -0.23224 | 0.89896 | 0.44198 |
| C49 | 0.07037 | 1.10014 | 0.54246 |
| C50 | 0.47749 | 1.38801 | 0.56344 |
| C51 | 0.51328 | 1.3692 | 0.58782 |
| C52 | 0.3687 | 1.38687 | 0.62727 |
| C53 | 0.19759 | 1.42475 | 0.64224 |
| C54 | 0.17258 | 1.44489 | 0.61868 |
| C55 | 0.30658 | 1.42545 | 0.57853 |
| C56 | 0.38688 | 1.36537 | 0.65229 |
| C57 | 0.38514 | 1.32134 | 0.63253 |
| C58 | 0.37575 | 1.30052 | 0.65561 |

| | | | |
|---|---|---|---|
| C59 | 0.37563 | 1.32458 | 0.69922 |
| C60 | 0.37587 | 1.3687 | 0.71986 |
| C61 | 0.3862 | 1.38865 | 0.696 |
| C62 | 0.34582 | 1.39393 | 0.76609 |
| C63 | 0.34659 | 1.25388 | 0.63437 |
| C64 | 0.16882 | 1.3785 | 0.78796 |
| C65 | 0.13657 | 1.40203 | 0.8314 |
| C66 | 0.26649 | 1.44275 | 0.85277 |
| C67 | 0.44145 | 1.45841 | 0.83144 |
| C68 | 0.48653 | 1.43395 | 0.78861 |
| C69 | 0.17333 | 1.23176 | 0.59694 |
| C70 | 0.14102 | 1.18791 | 0.5769 |
| C71 | 0.2683 | 1.16639 | 0.5961 |
| C72 | 0.4399 | 1.18799 | 0.63319 |
| C73 | 0.48414 | 1.23124 | 0.65181 |
| N74 | 0.22232 | 1.122 | 0.57742 |
| C75 | 0.06529 | 1.45495 | 0.91833 |
| N76 | 0.21998 | 1.46827 | 0.89673 |
| C77 | -0.01152 | 0.53372 | 0.38362 |
| C78 | 0.02479 | 0.5187 | 0.40974 |
| C79 | 0.0304 | 0.85811 | 0.48249 |
| C80 | 0.06826 | 0.89952 | 0.49762 |
| C81 | -0.0153 | 1.16258 | 0.53539 |
| C82 | -0.05242 | 1.12112 | 0.51999 |
| C83 | -0.0224 | 1.38552 | 0.85643 |
| C84 | -0.05986 | 1.41142 | 0.89748 |
| N85 | 0.06397 | 0.50669 | 0.34031 |
| C86 | 0.25156 | 0.51463 | 0.31547 |
| N87 | 0.27986 | 0.48126 | 0.27683 |
| C88 | 0.1037 | 0.45107 | 0.27783 |
| C89 | -0.02719 | 0.46643 | 0.31675 |
| N90 | 0.10799 | 0.84105 | 0.50893 |
| N91 | -0.08644 | 1.17981 | 0.50894 |
| C92 | 0.29153 | 0.80753 | 0.50046 |
| N93 | 0.3234 | 0.80166 | 0.53336 |
| C94 | 0.15341 | 0.83318 | 0.56378 |
| C95 | 0.02257 | 0.85753 | 0.54903 |
| C96 | -0.27215 | 1.21304 | 0.51705 |
| N97 | -0.29644 | 1.21929 | 0.4843 |
| C98 | -0.11956 | 1.18826 | 0.45435 |
| C99 | 0.00786 | 1.16383 | 0.46923 |
| N100 | -0.09413 | 1.34161 | 0.83936 |
| C101 | -0.27804 | 1.31652 | 0.80626 |
| N102 | -0.30181 | 1.2772 | 0.80003 |
| C103 | -0.12657 | 1.27802 | 0.83091 |
| C104 | -0.00062 | 1.3175 | 0.85524 |
| C105 | 0.13197 | 0.46981 | -0.00385 |
| C106 | 0.10074 | 0.49724 | 0.03915 |
| C107 | -0.03769 | 0.53741 | 0.05428 |
| C108 | -0.1468 | 0.5495 | 0.02562 |
| C109 | -0.11791 | 0.52177 | -0.01741 |
| C110 | 0.02387 | 0.48178 | -0.03254 |
| C111 | -0.07502 | 0.56582 | 0.09979 |
| N112 | -0.24498 | 0.53452 | -0.04545 |
| N113 | -0.29465 | 0.58981 | 0.03961 |
| N114 | 0.28493 | 0.43007 | -0.01743 |
| N115 | 0.2234 | 0.48409 | 0.06726 |
| N116 | -0.23837 | 0.55316 | 0.12132 |
| C117 | 0.06773 | 0.60841 | 0.12081 |
| C118 | 0.02982 | 0.63456 | 0.16181 |
| N119 | 0.10863 | 0.67828 | 0.17868 |
| C120 | 0.29055 | 0.70328 | 0.21204 |
| N121 | 0.32252 | 0.74236 | 0.21785 |

| | | | |
|---|---|---|---|
| C122 | 0.1544 | 0.74152 | 0.18647 |
| C123 | 0.02456 | 0.70225 | 0.16225 |
| C124 | 0.10381 | 1.47851 | 0.5652 |
| N125 | 0.26211 | 1.44376 | 0.5527 |
| C126 | -0.02411 | 1.5014 | 0.60816 |
| C127 | 0.01304 | 1.48649 | 0.63434 |
| N128 | -0.0612 | 1.51363 | 0.67768 |
| C129 | -0.24846 | 1.50584 | 0.70265 |
| N130 | -0.27537 | 1.53929 | 0.74127 |
| C131 | -0.09864 | 1.56936 | 0.74012 |
| C132 | 0.03124 | 1.55385 | 0.70112 |
| C133 | -0.30712 | 0.57842 | -0.03223 |
| C134 | -0.44704 | 0.60172 | 0.01163 |
| C135 | 0.28289 | 0.41126 | 0.00897 |
| C136 | 0.38052 | 0.44366 | 0.05322 |
| C137 | 0.27888 | 0.60695 | 0.60535 |
| C138 | 0.3611 | 0.61962 | 0.57307 |
| C139 | -0.27453 | 0.41125 | 0.41241 |
| C140 | -0.3577 | 0.39854 | 0.44464 |
| C141 | 0.28848 | 1.01242 | 0.414 |
| C142 | 0.38463 | 0.96765 | 0.40171 |
| C143 | -0.44482 | 1.0097 | 0.59984 |
| C144 | -0.30538 | 1.05402 | 0.62052 |
| H145 | 0.40036 | 0.54969 | 0.60439 |
| H146 | 0.20252 | 0.60359 | 0.51239 |
| H147 | -0.39846 | 0.46842 | 0.41327 |
| H148 | -0.20233 | 0.41481 | 0.5055 |
| H149 | -0.5677 | 0.53261 | 0.17018 |
| H150 | -0.64143 | 0.5752 | 0.24532 |
| H151 | -0.04787 | 0.67183 | 0.2474 |
| H152 | -0.38447 | 0.59814 | 0.30673 |
| H153 | -0.37111 | 0.7174 | 0.41942 |
| H154 | -0.35936 | 0.71199 | 0.30132 |
| H155 | -0.04891 | 0.77166 | 0.43402 |
| H156 | -0.55539 | 0.84963 | 0.37231 |
| H157 | -0.62869 | 0.77371 | 0.33927 |
| H158 | -0.08086 | 0.5822 | 0.34597 |
| H159 | -0.58027 | 0.64694 | 0.48536 |
| H160 | -0.64759 | 0.67986 | 0.44267 |
| H161 | -0.33669 | 0.94976 | 0.54875 |
| H162 | -0.26166 | 1.09934 | 0.60087 |
| H163 | 0.37571 | 1.06952 | 0.47123 |
| H164 | 0.23863 | 0.92135 | 0.41674 |
| H165 | 0.58354 | 1.37339 | 0.53275 |
| H166 | 0.65468 | 1.34081 | 0.57567 |
| H167 | 0.08364 | 1.43817 | 0.67212 |
| H168 | 0.38309 | 1.30321 | 0.59898 |
| H169 | 0.36738 | 1.3088 | 0.71714 |
| H170 | 0.38556 | 1.42253 | 0.71153 |
| H171 | 0.05769 | 1.3482 | 0.77095 |
| H172 | 0.54607 | 1.48945 | 0.84809 |
| H173 | 0.63313 | 1.44645 | 0.77328 |
| H174 | 0.0652 | 1.24891 | 0.58368 |
| H175 | 0.5421 | 1.17122 | 0.64749 |
| H176 | 0.62734 | 1.24684 | 0.6798 |
| H177 | 0.13421 | 0.48832 | 0.39858 |
| H178 | 0.18235 | 0.91833 | 0.52744 |
| H179 | -0.15608 | 1.10228 | 0.48951 |
| H180 | -0.16499 | 1.39952 | 0.91613 |
| H181 | 0.37089 | 0.54403 | 0.32509 |
| H182 | 0.07378 | 0.41967 | 0.25174 |
| H183 | -0.17893 | 0.44949 | 0.32704 |
| H184 | 0.40491 | 0.78762 | 0.471 |

| | | | |
|---|---|---|---|
| H185 | 0.12759 | 0.83816 | 0.59491 |
| H186 | -0.12531 | 0.88514 | 0.56627 |
| H187 | -0.39249 | 1.23246 | 0.54614 |
| H188 | -0.08673 | 1.18366 | 0.42345 |
| H189 | 0.15951 | 1.13654 | 0.45236 |
| H190 | -0.39729 | 1.32641 | 0.78689 |
| H191 | -0.09373 | 1.25142 | 0.83547 |
| H192 | 0.14967 | 1.32779 | 0.88241 |
| H193 | -0.26323 | 0.51332 | -0.07718 |
| H194 | -0.33521 | 0.6103 | 0.07104 |
| H195 | 0.36685 | 0.41175 | -0.04776 |
| H196 | 0.23123 | 0.50489 | 0.09916 |
| H197 | 0.18384 | 0.61959 | 0.10218 |
| H198 | 0.40263 | 0.69353 | 0.23191 |
| H199 | 0.12903 | 0.76796 | 0.18151 |
| H200 | -0.12188 | 0.69203 | 0.13477 |
| H201 | -0.13309 | 1.5318 | 0.61931 |
| H202 | -0.36847 | 1.47652 | 0.69315 |
| H203 | -0.06755 | 1.60076 | 0.76614 |
| H204 | 0.18325 | 1.57066 | 0.69071 |
| H205 | -0.47891 | 0.5803 | -0.05411 |
| H206 | -0.08183 | 0.59358 | -0.03368 |
| H207 | -0.41869 | 0.63604 | 0.02435 |
| H208 | -0.70505 | 0.59506 | 0.01059 |
| H209 | 0.45105 | 0.38425 | -0.00359 |
| H210 | 0.03884 | 0.39932 | 0.00897 |
| H211 | 0.31587 | 0.43165 | 0.07442 |
| H212 | 0.64296 | 0.44835 | 0.05483 |
| H213 | 0.44618 | 0.62245 | 0.63211 |
| H214 | 0.03175 | 0.6172 | 0.61705 |
| H215 | 0.62345 | 0.6179 | 0.56811 |
| H216 | 0.28498 | 0.65263 | 0.58496 |
| H217 | -0.02658 | 0.40121 | 0.40096 |
| H218 | -0.4401 | 0.39557 | 0.38548 |
| H219 | -0.2802 | 0.3656 | 0.43285 |
| H220 | -0.62029 | 0.40007 | 0.44939 |
| H221 | 0.45666 | 1.02507 | 0.39976 |
| H222 | 0.0442 | 1.01261 | 0.40233 |
| H223 | 0.64737 | 0.96577 | 0.40434 |
| H224 | 0.31699 | 0.94634 | 0.36874 |
| H225 | -0.41449 | 0.99685 | 0.62109 |
| H226 | -0.70333 | 1.0107 | 0.5945 |
| H227 | -0.08023 | 1.05556 | 0.63705 |
| H228 | -0.47746 | 1.0761 | 0.6442 |

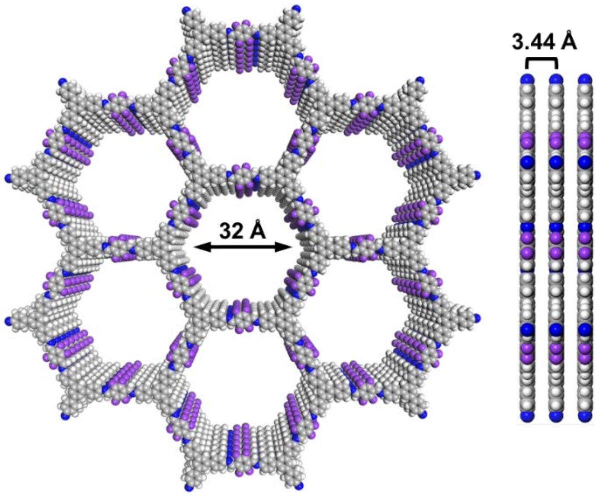


**Figure S1.** Top and side views of the eclipsed AA stacking crystal structure of COF-1. The C, N, F, and H atoms are represented by gray, blue, purple, and white spheres, respectively.

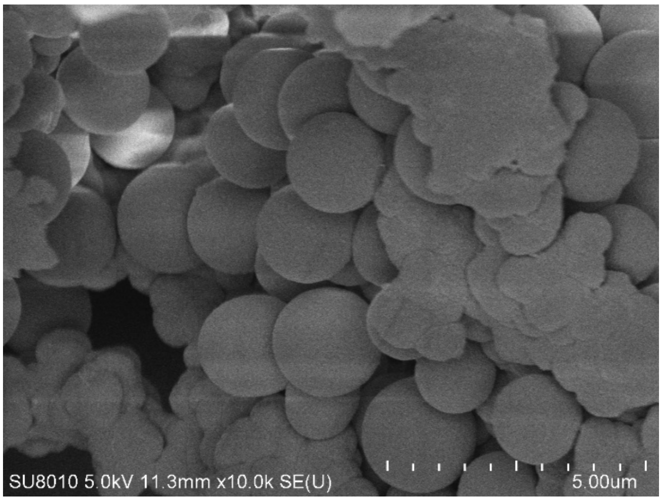


**Figure S2.** SEM image of COF-1.

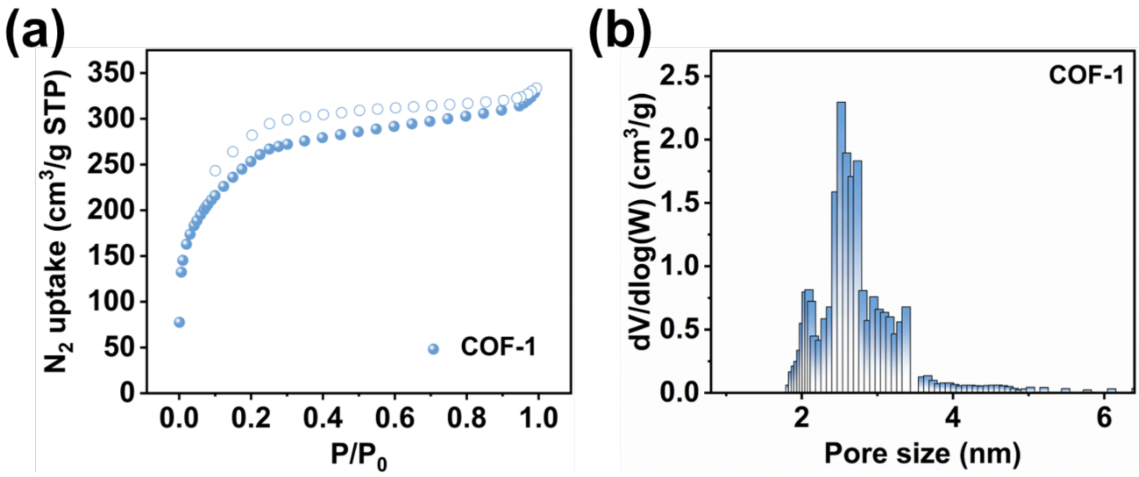


**Figure S3.** (a) $N_2$ sorption isotherms measured at 77 K for COF-1. (b) Pore size distribution of COF-1 determined by $N_2$ physisorption at 77 K.

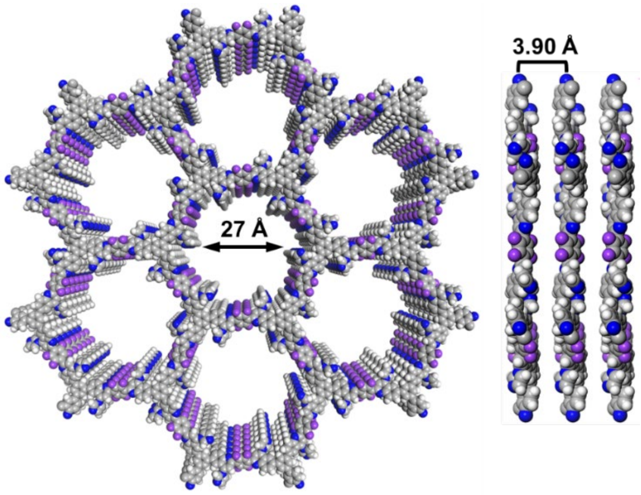


**Figure S4.** Top and side views of the eclipsed AA stacking crystal structure of COF-2. The C, N, F, and H atoms are represented by gray, blue, purple, and white spheres, respectively.

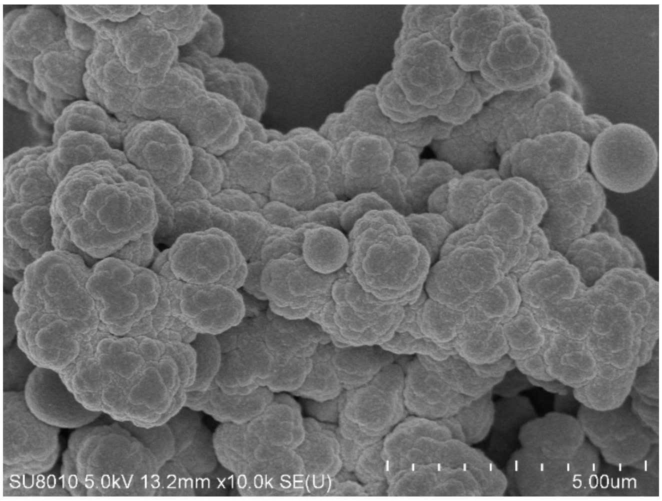


**Figure S5.** SEM image of COF-2.

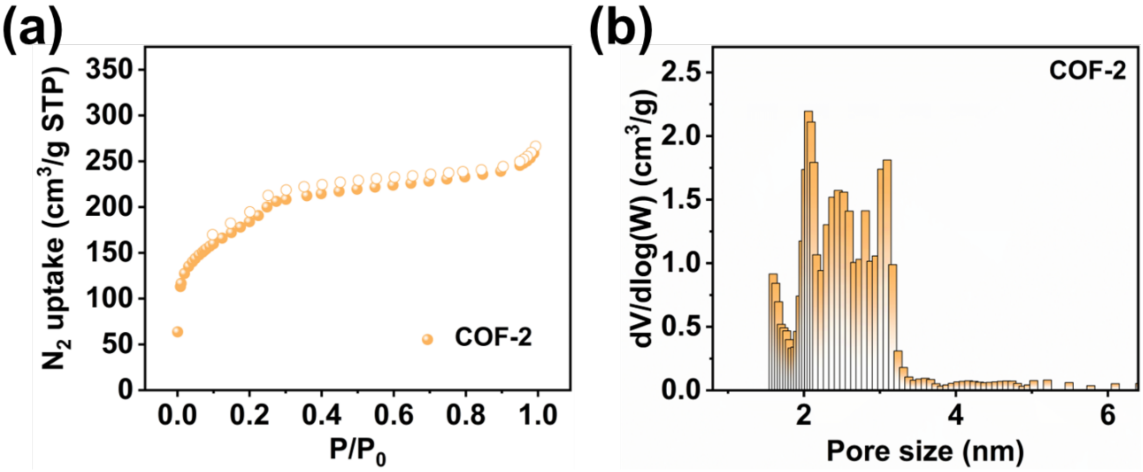


**Figure S6.** (a) $N_2$ sorption isotherms measured at 77 K for COF-2. (b) Pore size distribution of COF-2 determined by $N_2$ physisorption at 77 K.

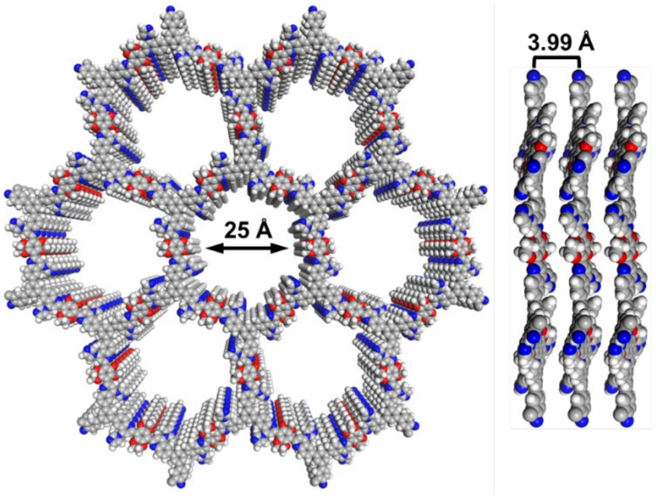


**Figure S7.** Top and side views of the eclipsed AA stacking crystal structure of COF-3O. The C, N, O, and H atoms are represented by gray, blue, red, and white spheres, respectively.

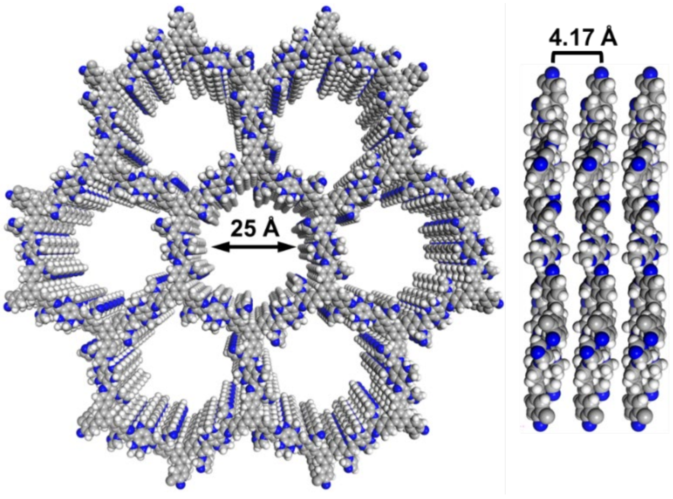


**Figure S8.** Top and side views of the eclipsed AA stacking crystal structure of COF-3N. The C, N, and H atoms are represented by gray, blue, and white spheres, respectively.

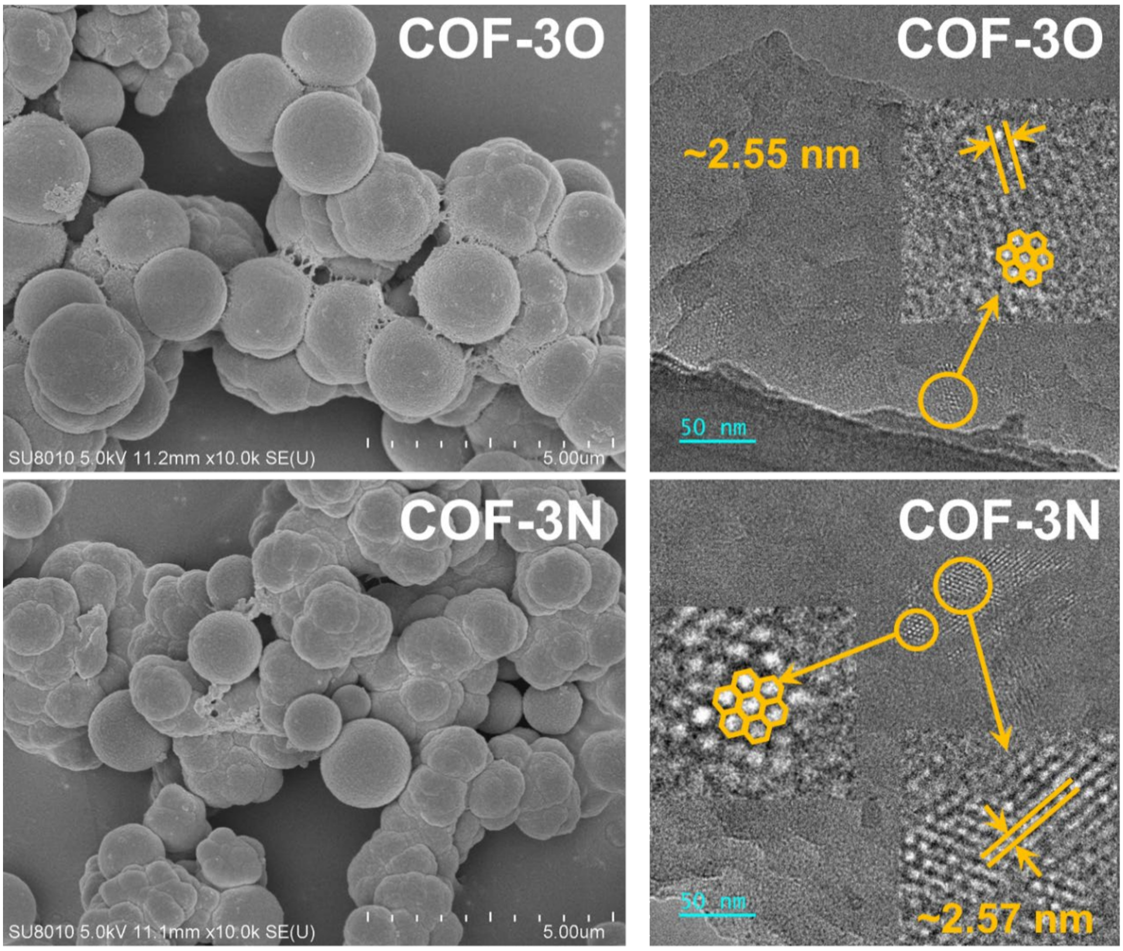


**Figure S9.** SEM (left) and TEM (right) images of COF-3O and COF-3N.

**Electrochemical measurements**

Electrochemical measurements were performed on a CHI760E electrochemical workstation. A standard three-electrode system was employed with a platinum wire and Ag/AgCl as the counter electrode and reference electrode, respectively. The working electrodes were prepared as follows: 5 mg of COF powder was mixed with 1 mL of ethanol and 10 μL of Nafion solution. Subsequently, the mixture was ultrasonicated for 30 min to achieve uniform mixing. Then, 0.1 mL of the suspension was applied dropwise on an indium-tin oxide (ITO) glass, then left to dry in air. The electrolyte was a 0.1 M $Na_2SO_4$ aqueous solution. The working electrodes were immersed in the electrolyte for 1 min before electrochemical measurement. Mott-Schottky plots were recorded at frequencies of 2000, 3000, and 4000 Hz. Electrochemical impedance spectroscopy (EIS) data were collected in the frequency range of 0.1-100000 Hz. The applied potential vs. Ag/AgCl was converted to a normal hydrogen electrode (NHE) potential using the following equation: $E_{NHE} = E_{Ag/AgCl} + E^{\theta}_{Ag/AgCl}$ ($E^{\theta}_{Ag/AgCl} = 0.199$ V).

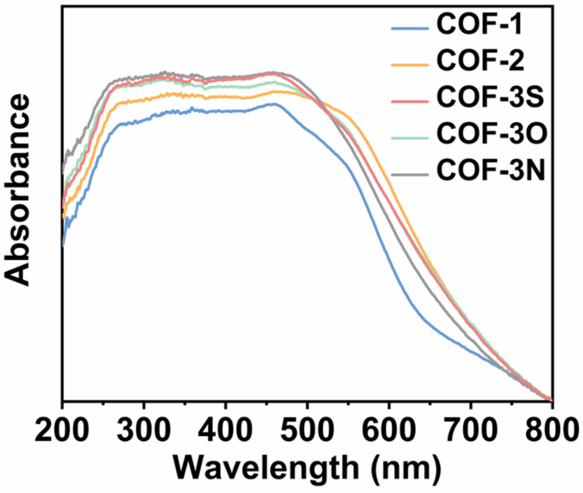


**Figure S10.** UV−visible diffuse reflectance spectra for all COFs.

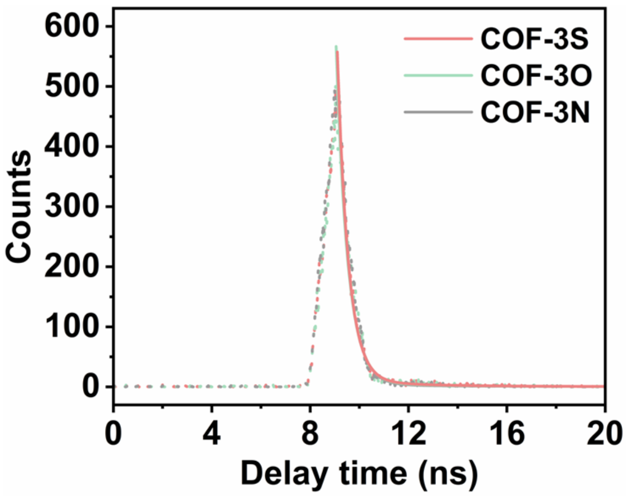


**Figure S11.** Photoluminescence decay curves for COF-3S, COF-3O, and COF-3N.

**Photocatalytic reduction of uranium studies**

Photocatalytic experiments were carried out at a fixed catalyst/liquid ratio of 0.05 g/L, under conditions of 25 °C and one standard sunlight (i.e., a solar irradiance of 1 kW/m²). The light source was a 300 W xenon lamp (PerfectLight, PLS-SXE300D, one standard sunlight). 5 mg of COF was thoroughly dispersed into 100 mL of a U(VI) solution (30 ppm in underground water and seawater). Then the reaction suspension was constantly stirred under dark conditions for 60 min to reach adsorption equilibrium, before irradiation with the Xe lamp. During the photocatalytic experiment, 1 mL aliquots of the reaction solution were taken at regular time intervals, filtered through a 0.22 μm polyether sulfone membrane, then the residual U(VI) concentration in the filtrate determined spectrophotometrically.

The residual U(VI) concentration was measured via UV-vis spectrophotometry at a wavelength of 650 nm using the Arsenazo III method. In detail, 1 mL of the filtered sample solution was thoroughly mixed with 4 mL of ultrapure water. Subsequently, $HNO_3$ (3M, 85 μL), 2,4-Dinitrophenol (1g/L, dissolved in ethanol/water (v/v, 2:8), 45 μL), chloroacetic acid-sodium acetate buffer solution (33.1 g chloroacetic acid and 12.3 g sodium acetate mixed in 1000 mL ultrapure water, 1 mL), and arsenazo III solution (0.5 g/L, 1 mL) were sequentially added to the mixture in turn. After mixing for ~10 min, the absorbance at 650 nm was measured.

**Uranium uptake capacity calculations.**

The U(VI) uptake capacity ($q_e$, mg/g) at equilibrium was calculated according to the following equation:

$$q_e = \frac{(C_0 - C_e) \times V}{m}$$

**Photocatalytic $H_2O_2$ production tests**

Briefly, COF photocatalyst (5 mg) was added to seawater and groundwater (50 mL) in a 100 mL beaker. The photocatalyst was dispersed by stirring under dark conditions for 30 min to reach adsorption-desorption equilibrium. The beaker was then irradiated at $\lambda > 420$ nm using a 300 W Xe lamp (PLS-SXE300, Beijing Perfectlight) under magnetic stirring at 25 ± 1 °C. At specific points in time, an aliquot of the solution was collected and filtered through a 0.22 μm filter to remove any photocatalyst. The concentration of $H_2O_2$ in the filtrate was then determined spectrophotometrically by the method described below.

**$H_2O_2$ detection tests**

The concentration of $H_2O_2$ was determined via iodometry according to previously reported studies.[1] Specifically, potassium iodide (KI) solution (1 mL, 0.4 mol/L) and potassium hydrogen phthalate ($C_8H_5KO_4$) solution (1 mL, 0.1 mol/L) were added to a diluted sample solution (0.5 mL) along with 2.5 mL of pure water, after which the resulting solution was left stirring for 30 min. $H_2O_2$ reacts with $I^-$ under acidic conditions to form $I_3^-$ ($H_2O_2 + 3I^- + 2H^+ \rightarrow I_3^- + 2H_2O$), which has a strong absorption maximum at about 350 nm by UV-vis spectroscopy. The total amount of $H_2O_2$ produced during the photocatalytic reaction was then calculated using a calibration graph of Absorbance at 350 nm versus $H_2O_2$ concentration, constructed using standard $H_2O_2$ solutions of known concentration.

In order to investigate the influence of different sacrificial agents on the $H_2O_2$ generation activity of all COFs, $AgNO_3$ (1 mM), $CH_3OH$ (10%), isopropyl alcohol (IPA, 10%) and p-benzoquinone (BQ, 5 mM) were used as radical scavengers for electrons ($e^-$), holes ($h^+$), ·OH, and $\cdot O_2^-$ radicals, respectively. To explore the influence of $O_2$ on the photocatalytic $H_2O_2$ generation activity, additional experiments were conducted by purging $N_2$ into the reaction solution under dark conditions for 30 min. Then, photocatalysis tests were carried out under a continuous $N_2$ flow. A 300 W Xe lamp (PLS-SXE300, Beijing Perfectlight) was used as the light source. The $H_2O_2$ generated in all these experiments was quantified spectrophotometrically.

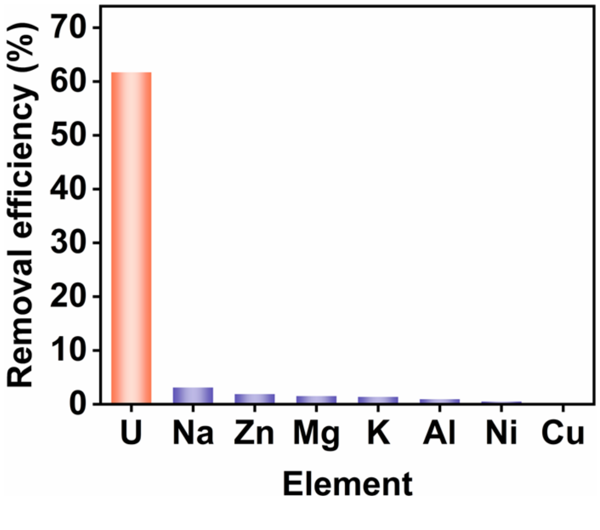


**Figure S12.** Selectivity of COF-3S for different metal cations.

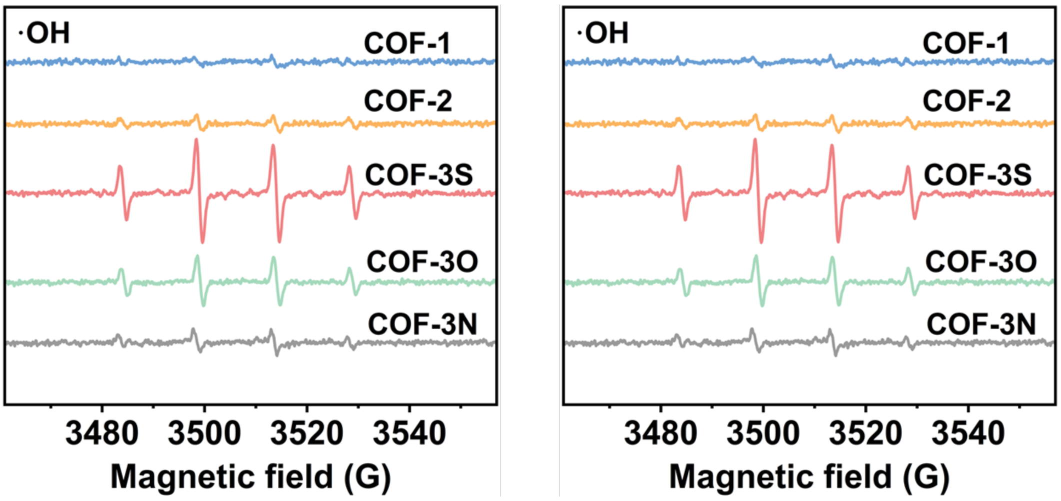


**Figure S13.** EPR spectra for (left) ·OH-DMPO and (right) $\cdot O_2^-$-DMPO adducts formed under visible light irradiation of COFs.

## Theoretical calculations

The geometries of the five different COFs were reduced to a simple repeating unit. These COF units and adsorption structures of COF-3S@$UO_2^{2+}$, COF-3S@$UO_2$, COF-3S@$(UO_2)O_2\cdot 2H_2O$, COF-3S@$UO_3\cdot H_2O$, and COF-3S@$UO_3$ were all optimized with dispersion corrected density functional theory (DFT-D3) at the PBE0-D3/def2-SVP39, 40 + SDD41-43 level using the Gaussian 16 program. Here, the SDD effective core potential was used to describe the atomic orbital and relativistic effect of the heavy element uranium. Vibrational frequency analyses were carried out for these optimized structures using the same calculation method to obtain zero-point and free energy corrections. In order to obtain the electron energy with high accuracy (which has a major impact on the accuracy of Gibbs free energy), single-point calculations for these optimized structures with PBE0-D3 functional and def2TZVP+SDD basis set were performed. Finally, the single point energy was added to the free energy correction calculated before to obtain the Gibbs free energy. The atomic dipole moment corrected Hirshfeld (ADCH) atomic charges, from which we can analyze the charge transfer directly, were obtained by population analysis from the wave function file of the DFT calculation using Multiwfn.[2] The adsorption free energy of the complex was calculated from the formula:

$$G(adsorb) = G(A+B) – G(A) – G(B)$$

where G(A) and G(B) are the Gibbs free energies of isolated molecules, and G(A+B) is the total free energy of the complex structure.

Subsequently, we use the Multiwfn program as a computational and analytical tool, combined with the Visual Molecular Dynamics program (VMD), to obtain the electrostatic potential distribution of different COFs.[3] The electron localization function (ELF) is mainly used to describe the spatial locality of electrons. The distribution characteristics of ELF can infer the strength of electrons bound by atoms. In this study, the planes containing heteroatoms and their adjacent benzene rings were selected to obtain the ELF spectra using the Multiwfn program.[4] The density of states (DOS) can directly or indirectly represent the degree of conjugation of material, and we can quantify the degree of conjugation by distinguishing the contribution of π and σ orbitals through the band structure and the projected density of states (PDOS). In this work, we select the heteroatoms and the benzene ring adjacent to the heteroatoms as fragments 1 and 2, and calculate their PDOS via Multiwfn to understand the conjugation degree of the COFs by judging the degree of their coincidence.

**Table S6**. The electrostatic potential distributions for COF-3S, COF-3O, and COF-3N

| | COF-3S | COF-3O | COF-3N |
|---|---|---|---|
| Charge (kcal/mol) | -46.38 | -47.60 | -47.52 |
| | -46.39 | -47.91 | -47.52 |

### Synthesis of COF-4S, COF-4O, and COF-4N

COF-1 (25 mg) and N,N-diisopropylethylamine (2 mL) were stirred in N,N-dimethylacetamide (DMAc, 5 mL, purged with $N_2$ for 10 min beforehand) in a 25 mL three-neck, round-bottom flask. Ethanedithiol (20 μL) was then injected under $N_2$ protection. The mixture was stirred at 70 °C for 8 h, after which a dark brown powder was obtained. After cooling to room temperature, the dark brown powder was collected by vacuum filtration and washed with ethanol, distilled water, and tetrahydrofuran (each three times). The powder was then dried at 45 °C for 12 h under vacuum, yielding COF-4S. COF-4O and COF-4N were prepared using the same protocol, replacing ethanedithiol with ethylene glycol (20 μL) and ethylenediamine (20 μL), and replacing diisopropylethylamine with potassium carbonate (49.75 mg) and cesium carbonate (117.29 mg), respectively.

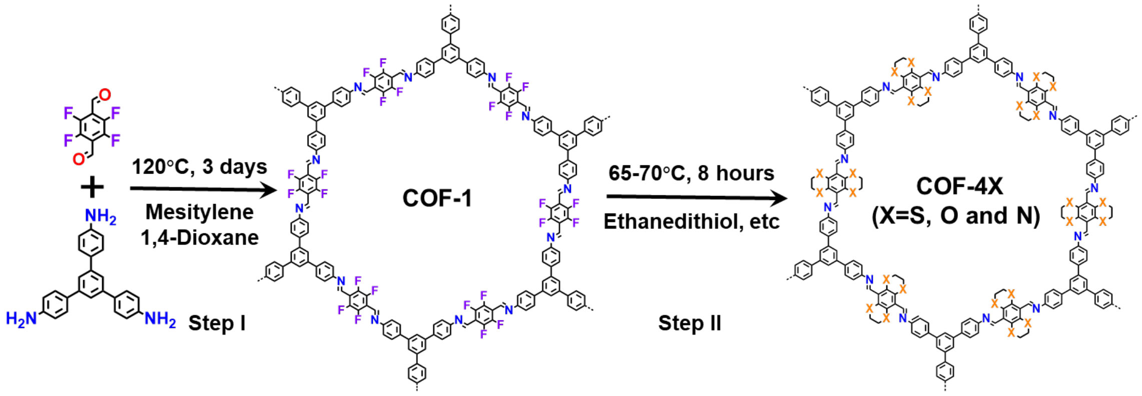


**Figure S14.** Illustration of the synthesis of COF-4S, COF-4O, and COF-4N.

**Table S7**. Fractional atomic coordinates for the AA-stacking unit cell of COF-4S

| space group P1, a = 37.12 Å, b = 37.25 Å; c = 4.18 Å, α =β = 90°, γ = 120° Pawley Refinement $R_p$ = 4.53%, $R_{wp}$ = 5.88% | | | |
|---|---|---|---|
| Atom | x (Å) | y (Å) | z (Å) |
| C1 | 0.54171 | 0.5453 | 0.37372 |
| C2 | 0.51315 | 0.55902 | 0.31624 |
| C3 | 0.47214 | 0.53628 | 0.43671 |
| C4 | 0.45897 | 0.49899 | 0.60709 |
| C5 | 0.48753 | 0.48527 | 0.66457 |
| C6 | 0.52854 | 0.50801 | 0.54415 |
| S7 | 0.59389 | 0.57033 | 0.19434 |
| S8 | 0.52692 | 0.60362 | 0.0627 |
| S9 | 0.40679 | 0.47397 | 0.78651 |
| S10 | 0.47376 | 0.44067 | 0.91814 |
| C11 | 0.55768 | 0.49206 | 0.58381 |
| C12 | 0.44303 | 0.55227 | 0.39743 |
| C13 | 0.1787 | 0.5807 | 0.32344 |
| C14 | 0.22208 | 0.60413 | 0.32358 |
| C15 | 0.24284 | 0.64143 | 0.49686 |
| C16 | 0.21908 | 0.65453 | 0.67126 |
| C17 | 0.17564 | 0.63119 | 0.66905 |
| C18 | 0.15508 | 0.59419 | 0.49151 |
| C19 | 0.28904 | 0.66659 | 0.49321 |
| C20 | 0.31315 | 0.64715 | 0.49278 |
| C21 | 0.35675 | 0.6705 | 0.4783 |
| C22 | 0.37626 | 0.71393 | 0.46913 |
| C23 | 0.35285 | 0.73407 | 0.47822 |
| C24 | 0.30923 | 0.71011 | 0.48777 |
| C25 | 0.37402 | 0.78009 | 0.47992 |
| C26 | 0.38186 | 0.64954 | 0.47388 |
| C27 | 0.41089 | 0.80345 | 0.65555 |
| C28 | 0.43083 | 0.84671 | 0.65937 |
| C29 | 0.41425 | 0.86753 | 0.48632 |
| C30 | 0.37726 | 0.8443 | 0.31528 |
| C31 | 0.3574 | 0.80105 | 0.30996 |
| C32 | 0.36834 | 0.61265 | 0.30021 |
| C33 | 0.39173 | 0.59268 | 0.29871 |
| C34 | 0.42917 | 0.60926 | 0.47234 |
| C35 | 0.44301 | 0.64635 | 0.64027 |
| C36 | 0.41957 | 0.66624 | 0.64313 |
| N37 | 0.45441 | 0.59005 | 0.48095 |
| C38 | 0.52445 | 0.00159 | 0.37057 |
| C39 | 0.53818 | 0.04389 | 0.3197 |
| C40 | 0.5151 | 0.06158 | 0.44283 |
| C41 | 0.4775 | 0.03689 | 0.60948 |
| C42 | 0.46377 | 1.00107 | 0.66035 |
| C43 | 0.48684 | 0.98337 | 0.53718 |
| S44 | 0.54996 | 0.98177 | 0.18652 |
| S45 | 0.58325 | 0.07545 | 0.07114 |
| S46 | 0.45198 | 0.06318 | 0.79353 |
| S47 | 0.4187 | 0.96951 | 0.90893 |
| C48 | 0.471 | 0.93834 | 0.57074 |
| N49 | 0.43329 | 0.91189 | 0.48252 |
| C50 | 0.53093 | 0.1066 | 0.40907 |
| C51 | 0.55794 | 0.39808 | 0.34168 |
| C52 | 0.58148 | 0.3783 | 0.33892 |
| C53 | 0.61919 | 0.3951 | 0.50838 |
| C54 | 0.63257 | 0.43195 | 0.68229 |
| C55 | 0.60908 | 0.4518 | 0.68368 |
| C56 | 0.57166 | 0.43515 | 0.50969 |
| C57 | 0.64445 | 0.37428 | 0.50384 |
| C58 | 0.62508 | 0.33087 | 0.51228 |
| C59 | 0.64865 | 0.31088 | 0.50325 |
| C60 | 0.69227 | 0.33499 | 0.49474 |

| | | | |
|---|---|---|---|
| C61 | 0.7123 | 0.3785 | 0.49012 |
| C62 | 0.68804 | 0.39779 | 0.49013 |
| C63 | 0.75849 | 0.40385 | 0.48784 |
| C64 | 0.62763 | 0.26486 | 0.50068 |
| C65 | 0.7825 | 0.39095 | 0.3144 |
| C66 | 0.82592 | 0.41451 | 0.31751 |
| C67 | 0.84621 | 0.45154 | 0.49481 |
| C68 | 0.82237 | 0.46482 | 0.66203 |
| C69 | 0.77901 | 0.44116 | 0.66125 |
| C70 | 0.59082 | 0.24146 | 0.32458 |
| C71 | 0.57098 | 0.19821 | 0.32034 |
| C72 | 0.58761 | 0.17744 | 0.49341 |
| C73 | 0.62457 | 0.20071 | 0.66471 |
| C74 | 0.64432 | 0.24396 | 0.67047 |
| N75 | 0.56863 | 0.13308 | 0.49719 |
| N76 | 0.54635 | 0.45428 | 0.50069 |
| C77 | 0.98792 | 0.4827 | 0.61774 |
| C78 | 0.02114 | 0.51021 | 0.65094 |
| C79 | 0.03876 | 0.55098 | 0.52973 |
| C80 | 0.01355 | 0.56405 | 0.37233 |
| C81 | 0.98037 | 0.53584 | 0.33251 |
| C82 | 0.96259 | 0.49533 | 0.45992 |
| C83 | 0.91745 | 0.46574 | 0.42994 |
| C84 | 0.08397 | 0.58026 | 0.56171 |
| S85 | 0.94843 | 0.54949 | 0.09037 |
| S86 | 0.03963 | 0.61543 | 0.18321 |
| S87 | 0.96473 | 0.43137 | 0.79471 |
| S88 | 0.05317 | 0.49603 | 0.89328 |
| N89 | 0.89068 | 0.47712 | 0.50455 |
| N90 | 0.11058 | 0.56889 | 0.48152 |
| C91 | 0.96648 | 0.59935 | 0.28385 |
| C92 | 1.00955 | 0.63125 | 0.1688 |
| C93 | 1.00198 | 0.41978 | 0.60326 |
| C94 | 0.03538 | 0.44262 | 0.75607 |
| C95 | 0.60883 | 0.62518 | 0.14644 |
| C96 | 0.57683 | 0.63654 | 0.25389 |
| C97 | 0.39185 | 0.41911 | 0.83446 |
| C98 | 0.42385 | 0.40775 | 0.72701 |
| C99 | 0.39677 | 0.02305 | 0.82766 |
| C100 | 0.38581 | 0.98648 | 0.71698 |
| C101 | 0.60517 | 0.01543 | 0.15239 |
| C102 | 0.61613 | 0.05847 | 0.26308 |
| H103 | 0.58943 | 0.51344 | 0.65266 |
| H104 | 0.41127 | 0.53093 | 0.32845 |
| H105 | 0.16331 | 0.55223 | 0.18646 |
| H106 | 0.23931 | 0.59345 | 0.18192 |
| H107 | 0.23405 | 0.68239 | 0.81537 |
| H108 | 0.15859 | 0.64181 | 0.81263 |
| H109 | 0.29796 | 0.61371 | 0.51055 |
| H110 | 0.4098 | 0.73213 | 0.44843 |
| H111 | 0.29095 | 0.7254 | 0.49107 |
| H112 | 0.42381 | 0.78823 | 0.79684 |
| H113 | 0.45844 | 0.86337 | 0.80437 |
| H114 | 0.36412 | 0.8599 | 0.18116 |
| H115 | 0.32947 | 0.7841 | 0.16786 |
| H116 | 0.34006 | 0.59965 | 0.15979 |
| H117 | 0.38075 | 0.56496 | 0.15592 |
| H118 | 0.47179 | 0.6595 | 0.77458 |
| H119 | 0.43056 | 0.69422 | 0.7835 |
| H120 | 0.49246 | 0.92811 | 0.64011 |
| H121 | 0.50944 | 0.11681 | 0.33981 |
| H122 | 0.52917 | 0.38487 | 0.20713 |
| H123 | 0.57059 | 0.35035 | 0.19838 |
| H124 | 0.66083 | 0.44501 | 0.82297 |

| | | | |
|---|---|---|---|
| H125 | 0.61997 | 0.47949 | 0.82663 |
| H126 | 0.59155 | 0.31255 | 0.5324 |
| H127 | 0.71066 | 0.31982 | 0.49165 |
| H128 | 0.70311 | 0.43122 | 0.47276 |
| H129 | 0.76772 | 0.36308 | 0.17024 |
| H130 | 0.8432 | 0.40409 | 0.1746 |
| H131 | 0.83757 | 0.49332 | 0.79878 |
| H132 | 0.7616 | 0.45168 | 0.80231 |
| H133 | 0.57784 | 0.25664 | 0.18334 |
| H134 | 0.54339 | 0.18151 | 0.17508 |
| H135 | 0.63774 | 0.18516 | 0.79883 |
| H136 | 0.67222 | 0.26094 | 0.8129 |
| H137 | 0.90731 | 0.43383 | 0.37041 |
| H138 | 0.09428 | 0.61201 | 0.62756 |
| H139 | 0.9445 | 0.61036 | 0.2233 |
| H140 | 0.96599 | 0.59577 | 0.54564 |
| H141 | 0.01119 | 0.6597 | 0.31568 |
| H142 | 1.00766 | 0.64005 | 0.92858 |
| H143 | 0.98984 | 0.38588 | 0.61917 |
| H144 | 1.00412 | 0.42777 | 0.34751 |
| H145 | 0.05863 | 0.44364 | 0.58375 |
| H146 | 0.03591 | 0.42456 | 0.96503 |
| H147 | 0.63789 | 0.6441 | 0.28435 |
| H148 | 0.61646 | 0.63341 | 0.9007 |
| H149 | 0.57337 | 0.63468 | 0.51626 |
| H150 | 0.58769 | 0.66911 | 0.18312 |
| H151 | 0.38423 | 0.41089 | 0.08022 |
| H152 | 0.36279 | 0.40019 | 0.69658 |
| H153 | 0.41298 | 0.37518 | 0.79781 |
| H154 | 0.4273 | 0.4096 | 0.46464 |
| H155 | 0.38747 | 0.02182 | 0.07131 |
| H156 | 0.37831 | 0.03338 | 0.68601 |
| H157 | 0.35303 | 0.96449 | 0.78363 |
| H158 | 0.38819 | 0.98537 | 0.4548 |
| H159 | 0.62363 | 0.0051 | 0.29404 |
| H160 | 0.61447 | 0.01666 | 0.90874 |
| H161 | 0.61376 | 0.05958 | 0.52526 |
| H162 | 0.64892 | 0.08046 | 0.19644 |

**Table S8**. Fractional atomic coordinates for the AA-stacking unit cell of COF-4O

| space group P-1, a = 37.94 Å, b = 36.07 Å; c = 3.88 Å, α = β = 90°, γ = 120° Pawley Refinement $R_p$ = 3.25%, $R_{wp}$ = 4.13% | | | |
|---|---|---|---|
| Atom | x (Å) | y (Å) | z (Å) |
| C1 | 0.52308 | 0.9802 | 0.57211 |
| C2 | 0.54216 | 0.02349 | 0.49109 |
| C3 | 0.51898 | 0.0436 | 0.42084 |
| O4 | 0.54593 | 0.96 | 0.64923 |
| O5 | 0.58424 | 0.0466 | 0.45977 |
| C6 | 0.46171 | 0.91031 | 0.63389 |
| C7 | 0.56544 | 0.38139 | 0.95117 |
| C8 | 0.5899 | 0.36224 | 0.95926 |
| C9 | 0.62816 | 0.38202 | 0.80522 |
| C10 | 0.64198 | 0.42222 | 0.65712 |
| C11 | 0.61768 | 0.44149 | 0.65234 |
| C12 | 0.57887 | 0.42089 | 0.79586 |
| C13 | 0.65237 | 0.35973 | 0.77316 |
| C14 | 0.63347 | 0.31609 | 0.70726 |
| C15 | 0.65604 | 0.29524 | 0.64501 |
| C16 | 0.69819 | 0.31885 | 0.64674 |
| C17 | 0.71767 | 0.36235 | 0.71796 |
| C18 | 0.6945 | 0.38235 | 0.78561 |
| C19 | 0.76218 | 0.38722 | 0.70801 |
| C20 | 0.63557 | 0.24885 | 0.58109 |
| C21 | 0.78606 | 0.37056 | 0.84219 |
| C22 | 0.82796 | 0.3934 | 0.82013 |
| C23 | 0.84677 | 0.43351 | 0.66246 |
| C24 | 0.82295 | 0.45035 | 0.53527 |
| C25 | 0.78114 | 0.42753 | 0.55656 |
| C26 | 0.59871 | 0.22876 | 0.40394 |
| C27 | 0.57809 | 0.18483 | 0.36668 |
| C28 | 0.59418 | 0.16001 | 0.50387 |
| C29 | 0.63147 | 0.18009 | 0.66963 |
| C30 | 0.65194 | 0.22393 | 0.70948 |
| N31 | 0.57322 | 0.1146 | 0.49853 |
| C32 | 0.98485 | 0.45675 | 0.56502 |
| C33 | 0.02545 | 0.48201 | 0.46446 |
| C34 | 0.04048 | 0.52545 | 0.39787 |
| O35 | 0.96954 | 0.41337 | 0.63188 |
| O36 | 0.05046 | 0.46367 | 0.41259 |
| C37 | 0.9166 | 0.44702 | 0.6936 |
| N38 | 0.88949 | 0.45782 | 0.61516 |
| C39 | 0.45953 | 0.46959 | 0.45122 |
| C40 | 0.48808 | 0.45703 | 0.55054 |
| C41 | 0.52861 | 0.48766 | 0.60204 |
| C42 | 0.43984 | 0.52464 | 0.31531 |
| O43 | 0.52376 | 0.58616 | 0.39372 |
| O44 | 0.58066 | 0.561 | 0.60784 |
| N45 | 0.44898 | 0.56344 | 0.24543 |
| C46 | 0.56158 | 0.61585 | 0.5362 |
| C47 | 0.59312 | 0.60202 | 0.46787 |
| C48 | 0.58637 | 0.98072 | 0.52695 |
| C49 | 0.60649 | 0.02779 | 0.6047 |
| C50 | 0.00774 | 0.60562 | 0.50628 |
| C51 | 0.96284 | 0.57744 | 0.44078 |
| H52 | 0.47889 | 0.89742 | 0.75833 |
| H53 | 0.53559 | 0.36507 | 0.06149 |
| H54 | 0.5786 | 0.33171 | 0.08223 |
| H55 | 0.67075 | 0.43788 | 0.52735 |
| H56 | 0.62795 | 0.46966 | 0.52603 |
| H57 | 0.60103 | 0.29836 | 0.70048 |
| H58 | 0.71592 | 0.3036 | 0.58255 |

| | | | |
|---|---|---|---|
| H59 | 0.70938 | 0.41569 | 0.84804 |
| H60 | 0.77231 | 0.33995 | 0.96684 |
| H61 | 0.84502 | 0.37935 | 0.92751 |
| H62 | 0.83693 | 0.48101 | 0.41141 |
| H63 | 0.76362 | 0.44096 | 0.44513 |
| H64 | 0.58573 | 0.24696 | 0.29248 |
| H65 | 0.54972 | 0.17094 | 0.23221 |
| H66 | 0.64411 | 0.16143 | 0.77899 |
| H67 | 0.6798 | 0.23806 | 0.85325 |
| H68 | 0.90812 | 0.41646 | 0.80499 |
| H69 | 0.40866 | 0.50054 | 0.32428 |
| H70 | 0.57121 | 0.64735 | 0.41915 |
| H71 | 0.55819 | 0.61864 | 0.81603 |
| H72 | 0.62213 | 0.62486 | 0.58667 |
| H73 | 0.59809 | 0.60148 | 0.18803 |
| H74 | 0.60336 | 0.96653 | 0.65241 |
| H75 | 0.58644 | 0.97541 | 0.24683 |
| H76 | 0.60904 | 0.03319 | 0.88537 |
| H77 | 0.63735 | 0.04355 | 0.49634 |
| H78 | 0.01351 | 0.6107 | 0.78579 |
| H79 | 0.01791 | 0.63697 | 0.38452 |
| H80 | 0.94559 | 0.59195 | 0.55617 |
| H81 | 0.95675 | 0.57423 | 0.16123 |

**Table S9**. Fractional atomic coordinates for the AA-stacking unit cell of COF-4N

| space group P-1, a = 37.37 Å, b = 37.35 Å; c = 3.95 Å, α = β = 90°, γ = 120° Pawley Refinement $R_p$ = 3.46%, $R_{wp}$ = 4.56% | | | |
|---|---|---|---|
| Atom | x (Å) | y (Å) | z (Å) |
| C1 | 0.54395 | 0.52639 | 0.4084 |
| C2 | 0.51587 | 0.54048 | 0.34941 |
| C3 | 0.47404 | 0.51614 | 0.44346 |
| N4 | 0.5862 | 0.55147 | 0.2948 |
| N5 | 0.53088 | 0.57957 | 0.1822 |
| C6 | 0.5591 | 0.47187 | 0.60694 |
| C7 | 0.18122 | 0.55897 | 0.38713 |
| C8 | 0.22431 | 0.58205 | 0.3896 |
| C9 | 0.24497 | 0.62039 | 0.55676 |
| C10 | 0.22128 | 0.6348 | 0.72306 |
| C11 | 0.17809 | 0.61176 | 0.71768 |
| C12 | 0.15771 | 0.57365 | 0.54665 |
| C13 | 0.2909 | 0.64538 | 0.55 |
| C14 | 0.31473 | 0.62584 | 0.55338 |
| C15 | 0.35787 | 0.64871 | 0.52272 |
| C16 | 0.37734 | 0.69188 | 0.49799 |
| C17 | 0.35443 | 0.71235 | 0.51055 |
| C18 | 0.31116 | 0.68874 | 0.53249 |
| C19 | 0.37585 | 0.7583 | 0.50406 |
| C20 | 0.38258 | 0.62749 | 0.51584 |
| C21 | 0.41399 | 0.78178 | 0.66937 |
| C22 | 0.4342 | 0.82493 | 0.66697 |
| C23 | 0.41654 | 0.84563 | 0.49964 |
| C24 | 0.37849 | 0.82235 | 0.33746 |
| C25 | 0.35833 | 0.77917 | 0.33761 |
| C26 | 0.3676 | 0.58934 | 0.34971 |
| C27 | 0.39069 | 0.56922 | 0.34517 |
| C28 | 0.42938 | 0.58701 | 0.50679 |
| C29 | 0.44443 | 0.62508 | 0.66903 |
| C30 | 0.42135 | 0.64518 | 0.67521 |
| N31 | 0.45447 | 0.56782 | 0.51502 |
| C32 | 0.52648 | 0.98615 | 0.40623 |
| C33 | 0.54079 | 0.0248 | 0.34973 |
| C34 | 0.51667 | 0.04249 | 0.44615 |
| N35 | 0.55139 | 0.9688 | 0.29078 |
| N36 | 0.57985 | 0.04872 | 0.18214 |
| C37 | 0.47189 | 0.9166 | 0.60409 |
| N38 | 0.4357 | 0.88988 | 0.48782 |
| C39 | 0.98649 | 0.45969 | 0.58813 |
| C40 | 0.02421 | 0.48745 | 0.65787 |
| C41 | 0.04217 | 0.52959 | 0.57139 |
| C42 | 0.91777 | 0.44534 | 0.37168 |
| N43 | 0.95669 | 0.53194 | 0.17631 |
| N44 | 0.03545 | 0.58647 | 0.30697 |
| N45 | 0.89066 | 0.4548 | 0.47856 |
| C46 | 0.96898 | 0.57532 | 0.22008 |
| C47 | 0.01091 | 0.60254 | 0.14659 |
| C48 | 0.60159 | 0.59203 | 0.13569 |
| C49 | 0.57432 | 0.61078 | 0.21672 |
| C50 | 0.41225 | 0.00979 | 0.87127 |
| C51 | 0.3933 | 0.96742 | 0.78891 |
| H52 | 0.6061 | 0.5394 | 0.29236 |
| H53 | 0.5105 | 0.58716 | 0.06377 |
| H54 | 0.58875 | 0.49209 | 0.72086 |
| H55 | 0.16582 | 0.52988 | 0.25251 |
| H56 | 0.24134 | 0.57043 | 0.25062 |
| H57 | 0.23608 | 0.66348 | 0.86369 |
| H58 | 0.16119 | 0.62373 | 0.85242 |

| | | | |
|---|---|---|---|
| H59 | 0.29967 | 0.59268 | 0.58503 |
| H60 | 0.41044 | 0.70962 | 0.46243 |
| H61 | 0.2932 | 0.70418 | 0.53521 |
| H62 | 0.42773 | 0.76675 | 0.80951 |
| H63 | 0.46296 | 0.84149 | 0.80293 |
| H64 | 0.36485 | 0.83799 | 0.20539 |
| H65 | 0.32955 | 0.76222 | 0.20044 |
| H66 | 0.33844 | 0.57549 | 0.216 |
| H67 | 0.37826 | 0.54037 | 0.2101 |
| H68 | 0.47397 | 0.63879 | 0.79664 |
| H69 | 0.43338 | 0.674 | 0.8123 |
| H70 | 0.53917 | 0.93684 | 0.28644 |
| H71 | 0.58754 | 0.07678 | 0.0649 |
| H72 | 0.49217 | 0.90716 | 0.71712 |
| H73 | 0.90867 | 0.41567 | 0.2588 |
| H74 | 0.92931 | 0.51214 | 0.04812 |
| H75 | 0.0674 | 0.60615 | 0.30985 |
| H76 | 0.95061 | 0.5838 | 0.05151 |
| H77 | 0.96255 | 0.58037 | 0.48422 |
| H78 | 0.02137 | 0.63433 | 0.24015 |
| H79 | 0.01614 | 0.60444 | 0.87338 |
| H80 | 0.63353 | 0.61345 | 0.22476 |
| H81 | 0.6029 | 0.5887 | 0.86194 |
| H82 | 0.58018 | 0.62264 | 0.47973 |
| H83 | 0.58205 | 0.63738 | 0.04724 |
| H84 | 0.41558 | 0.01431 | 0.14509 |
| H85 | 0.39096 | 0.02055 | 0.78284 |
| H86 | 0.36676 | 0.94864 | 0.95879 |
| H87 | 0.38134 | 0.96158 | 0.52627 |

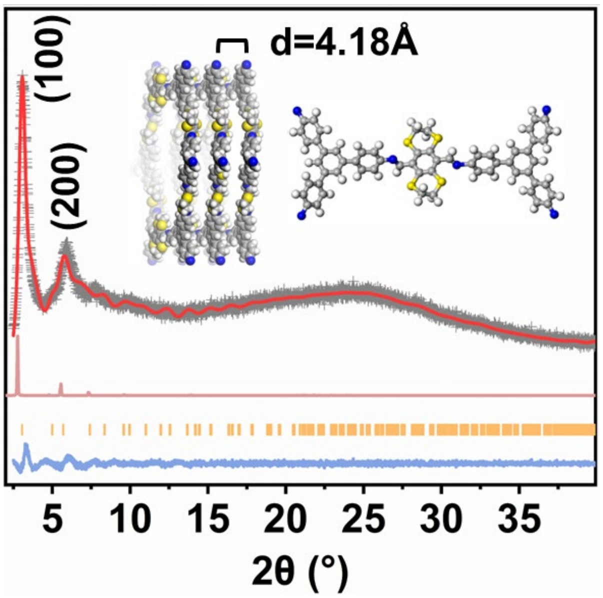


**Figure S15.** Experimental PXRD patterns of COF-4S with corresponding Pawley refinement (red), simulated results (light red), and Bragg positions (orange) showing a good fit for the experimental data (gray) with minimal differences (blue). The inset shows the structural model of COF-4S assuming the eclipsed (AA) stacking mode.

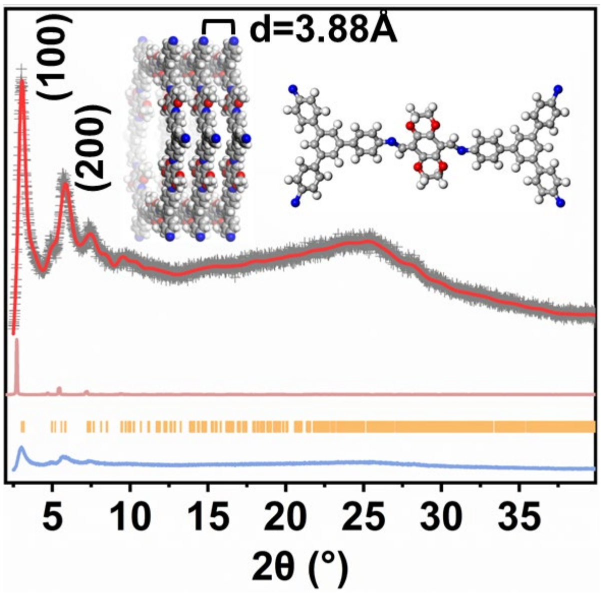


**Figure S16.** Experimental PXRD patterns of COF-4O with corresponding Pawley refinement (red), simulated results (light red), and Bragg positions (orange) showing a good fit for the experimental data (gray) with minimal differences (blue). The inset shows the structural model of COF-4O assuming the eclipsed (AA) stacking mode.

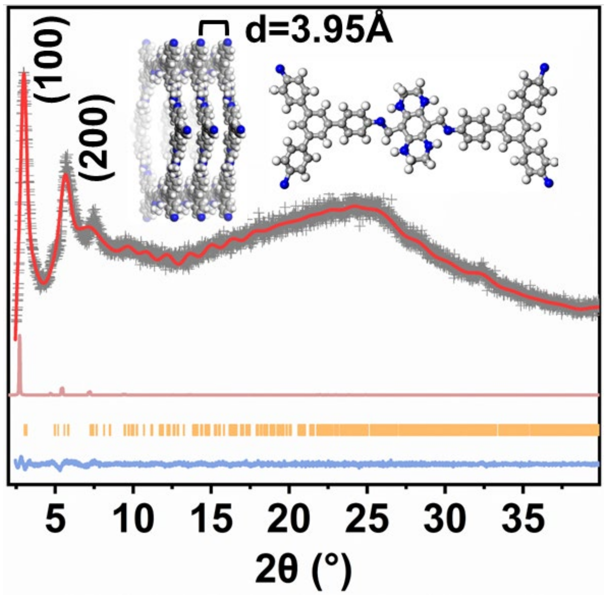


**Figure S17.** Experimental PXRD patterns of COF-4N with corresponding Pawley refinement (red), simulated results (light red), and Bragg positions (orange) showing a good fit for the experimental data (gray) with minimal differences (blue). The inset shows the structural model of COF-4N assuming the eclipsed (AA) stacking mode.

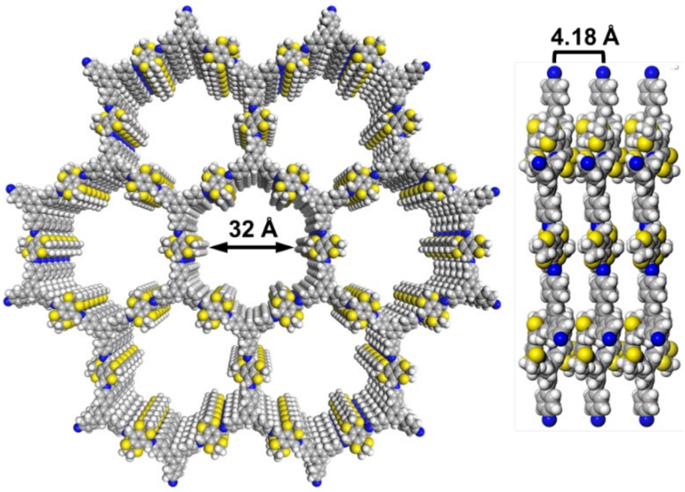


**Figure S18.** Top and side views of the eclipsed AA stacking crystal structure of COF-4S. The C, N, S, and H atoms are represented by gray, blue, yellow, and white spheres, respectively.

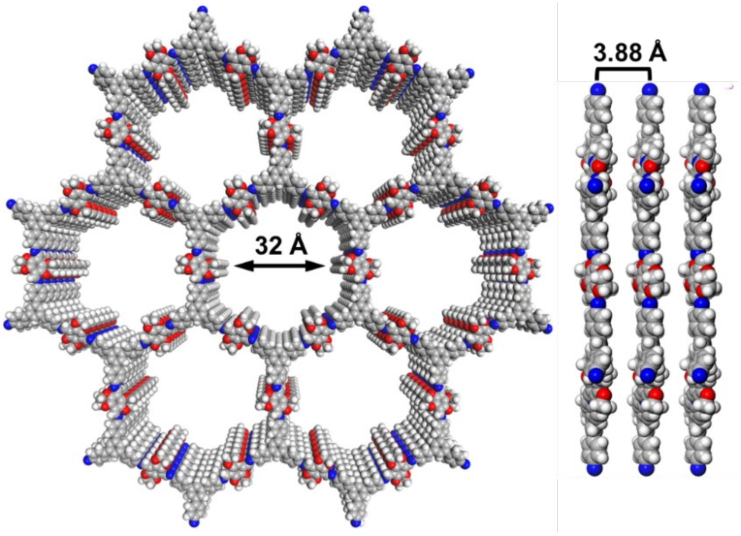


**Figure S19.** Top and side views of the eclipsed AA stacking crystal structure of COF-4O. The C, N, O, and H atoms are represented by gray, blue, red, and white spheres, respectively.

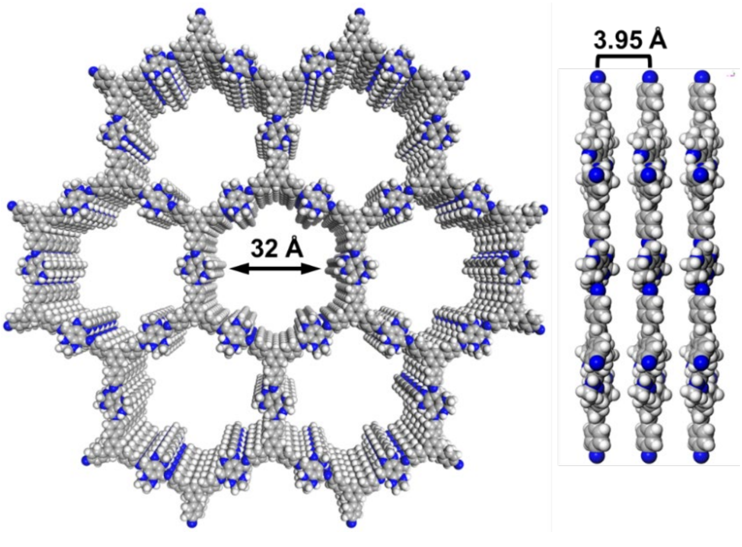


**Figure S20.** Top and side views of the eclipsed AA stacking crystal structure of COF-4N. The C, N, and H atoms are represented by gray, blue, and white spheres, respectively.

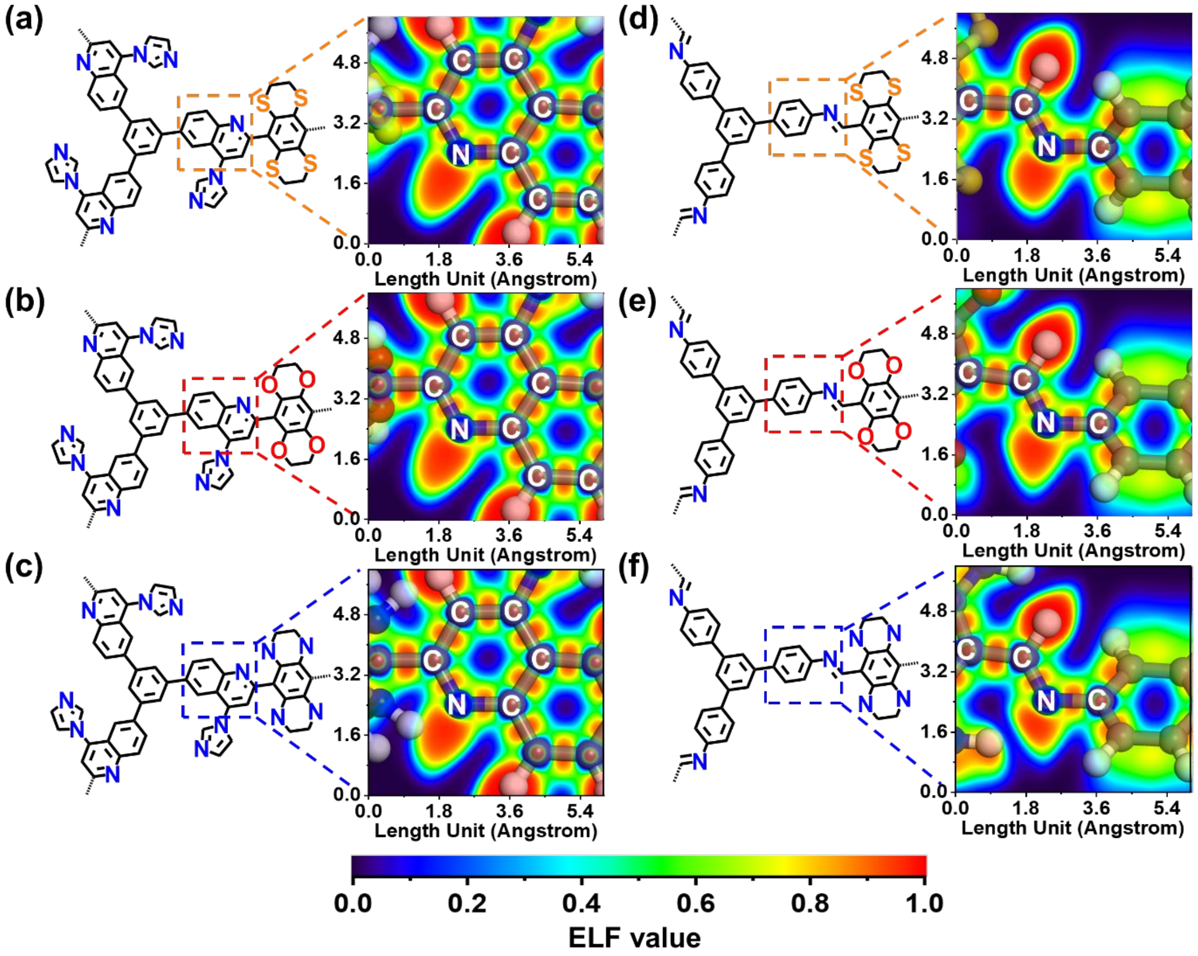


**Figure S21.** (a-c) The ELF diagrams of nitrogen atoms at quinoline rings of COF-3S, COF-3O, and COF-3N. (d-f) The ELF diagrams of nitrogen atoms at N=C groups of COF-4S, COF-4O, and COF-4N.

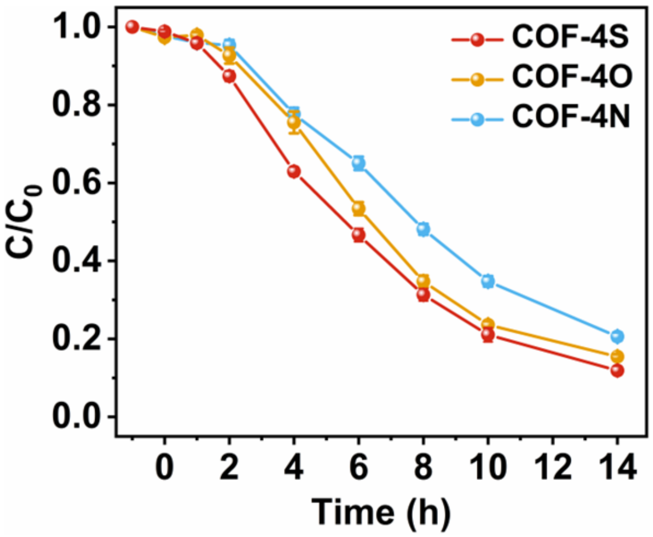


**Figure S22.** Uranium extraction from uranyl-spiked groundwater with initial uranyl concentrations of 30 ppm, using COF-4S, COF-4O, and COF-4N as photocatalysts.

## References

1. Wei, Z.; Liu, M.; Zhang, Z.; Yao, W.; Tan, H.; Zhu, Y., Efficient Visible-Light-Driven Selective Oxygen Reduction to Hydrogen Peroxide by Oxygen-Enriched Graphitic Carbon Nitride Polymers. *Energy Environ. Sci.* **2018,** *11* (9), 2581-2589.
2. Lu, T.; Chen, F. W., Multiwfn: A Multifunctional Wavefunction Analyzer. *J. Comput. Chem.* **2012,** *33* (5), 580-592.
3. Humphrey, W.; Dalke, A.; Schulten, K., VMD: Visual molecular dynamics. *J. Mol. Graph. Model.* **1996,** *14* (1), 33-38.
4. Lu, T.; Chen, F. W., Meaning and Functional Form of the Electron Localization Function. *Acta Phys. -Chim. Sin.* **2011,** *27* (12), 2786-2792.
