# Supplementary material for: Systematic Tuning of the Electronic Effects in Covalent Organic Frameworks for Promoting Photocatalysis
Source: ACS Cent Sci. 2025 Nov 18;11(12):2448–59. doi: 10.1021/acscentsci.5c01645 (PMC12746150; doi:10.1021/acscentsci.5c01645)
Supplement: Supplementary file 2 [file oc5c01645_si_002.pdf]

oc-2025-01645w.R1

Name: Peer Review Information for "Systematic Tuning of the Electronic Effects in Covalent Organic Frameworks for Promoting Photocatalysis"

First Round of Reviewer Comments

Reviewer: 1

Comments to the Author

This work proposed an interesting strategy for developing a series of N (S and O) atom doping COFs to regulate conjugation and induction effects, and their photocatalytic performance were compared. The authors demonstrated that the photocatalytic activity of covalent organic frameworks does not only depend on conjugation effect, also induction effect, which is often overlooked. This work, in my opinion, is novel, logically organized, and fully discussed. By combining experiments with theoretical calculations, they have obtained sufficient evidence to demonstrate the importance of balancing conjugation and induction effects. This manuscript can be considered to publish in ACS Central Science. The results seem encouraging and characterization are relatively decent, however, before publication, some objectives should be first taken into account.Comments:1. In the article, the induction effect (or inductive effect) seems to have multiple forms of expression. Please clarify and standardize the expression.2. Regarding Figure 1b, what do the red and blue areas represent respectively? The shades of colors are also different. Please provide more explanations in your reply.3. Why didn't the author attempt to directly synthesize COF-3X by solvothermal method? Would applying two consecutive modifications cause significant and irreversible structural damage to the materials?4. In Fig.3b, the experimental data (gray) seems incomplete. The authors are requested to confirm whether the data is complete and to provide data ranging from 2 to 40°.5. Which specific position does the PDOS segments in Figure 6 refer to? In my opinion, it is necessary to insert the schematic diagrams of each fragment in Figures 6g, h, and i.

Reviewer: 2

Comments to the Author

In this manuscript, Ma and co-workers designed and prepared a series of heteroatom-doped COFs, aiming to investigate the relative importance of conjugation and induction effects in photocatalytic reactivity. The concept stands out among numerous works related to COFs, specifically in terms of the proposal of the induction effect. Even for highly conjugated COF, if it does not possess electron-rich regions such as heteroatoms (N, O, or S), the photocatalytic performance seems unlikely to be particularly outstanding. In the previous works focused on the study of COFs as photocatalysts, researchers tend to concentrate solely on the conjugation effect, while ignoring the induction effect. Crucially, both induction and conjugation effects belong to the electronic effects, which determine the electron transfer process within the COF photocatalysts. The manuscript is well-organized, exhibits high novelty, and provides logical results that sufficiently support the conclusions. Meanwhile, the proposed strategies have certain universality, which is expected to draw the broad interests of researchers. Therefore, this manuscript meets the standards required by ACS Central Science, and it is recommended for publication after minor revision.

Special comments:

1. Can COF-3S, COF-3O, and COF-3N be directly synthesized? If so, there is no need to prepare COF-2. It is necessary to state in revised form.

2. The authors mentioned “Subsequently, post-synthetic modification transformed imine bonds to chemically stable chromenoquinoline rings through the Povarov reaction, increasing π-conjugation.” If the purpose of the Povarov reaction is to increase π conjugation, it seems that it does not make a significant contribution to the exploration of the mechanism. It is suggested that the core purpose of this step be explained in the Introduction.

3. In the Introduction, “one of our developed COF-3S delivered a high photocatalytic uranium removal efficiency in contaminated groundwater and tap water.”, there seems to be a mistake in this sentence. It should be seawater and groundwater. Please check it carefully.

4. The authors used post-modification technique to synthesize COF-3S, COF-3O and COF-3N. Is it possible to synthesize these COFs using solvothermal techniques directly?

5. It is better to describe the conditions for the equilibrium state of conjugation and induction effects in photocatalyst.

Author's Response to Peer Review Comments:

Date: October 22, 2025

Senior Editor
ACS Central Science

Dear Editor:

Thank you very much for the opportunity to revise our manuscript titled “***Systematic Tuning of the Electronic Effects in Covalent Organic Frameworks for Promoting Photocatalysis***” (Manuscript ID: oc-2025-01645w). We greatly appreciate the constructive comments and suggestions from all reviewers, and we have revised our manuscript accordingly, as detailed in the responses below. The corresponding changes have been highlighted in yellow in the main text and supporting information.

Reviewers' comments:

<u>Reviewer #1:</u>

This work proposed an interesting strategy for developing a series of N (S and O) atom doping COFs to regulate conjugation and induction effects, and their photocatalytic performance were compared. The authors demonstrated that the photocatalytic activity of covalent organic frameworks does not only depend on conjugation effect, also induction effect, which is often overlooked. This work, in my opinion, is novel, logically organized, and fully discussed. By combining experiments with theoretical calculations, they have obtained sufficient evidence to demonstrate the importance of balancing conjugation and induction effects. This manuscript can be considered to publish in ACS Central Science. The results seem encouraging and characterization are relatively decent, however, before publication, some objectives should be first taken into account.

Response: We are grateful to the reviewer for taking the time to evaluate our work and greatly appreciate their positive comments and support.

Comment 1: In the article, the induction effect (or inductive effect) seems to have multiple forms of expression. Please clarify and standardize the expression.

Response: We greatly appreciate the constructive suggestions from the reviewer. In the manuscript, the term "induction effect" is used correctly. We have conducted a thorough review of the entire manuscript and standardized all terminology to "induction effect".

Comment 2: Regarding Figure 1b, what do the red and blue areas represent respectively? The shades of colors are also different. Please provide more explanations in your reply.

Response: We appreciate the comment from the reviewer. In Figure 1b, the red area denotes the electron-deficient region, while the blue area signifies the electron-rich region. Additionally, the intensity of color reflects varying degrees of electron deficiency or abundance. In this manuscript, we discuss conjugation and induction effects. As the electronegativity of heteroatoms within the materials varies, so too does the strength of

these induction effects, resulting in changes to electron distribution. As illustrated in Figure 1b, with a gradual increase in electronegativity among heteroatoms (in the order of O, S, and N), there is a corresponding intensification of induction effects; consequently, differences in electron deficiency and richness within the microstructure become increasingly pronounced. These variations ultimately manifest as differences in both the extent of red and blue areas and fluctuations in color intensity.

Comment 3: Why didn't the author attempt to directly synthesize COF-3X by solvothermal method? Would applying two consecutive modifications cause significant and irreversible structural damage to the materials?

Response: We appreciate the comment from the reviewer. We have endeavored to directly synthesize COF-3X through the solvothermal method during the material preparation process. Following the ligand synthesis techniques proposed by Ye et al., we successfully synthesized 2,3,7,8tetrahydrobenzo[1,2-b:4,5-b']bis([1,4]dithiine)-5,10-dicarbaldehyde (referred to as S2 in Ye et al.'s work published in Nat. Commun. 2022, 13 (1), 6116).

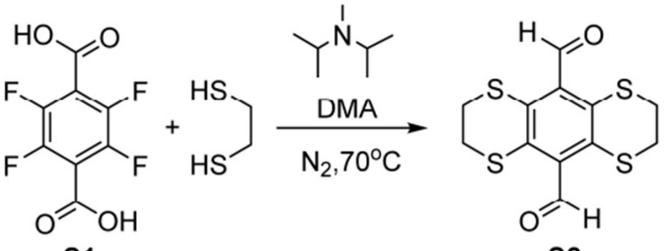

S1 S2

The synthetic scheme for S2

Subsequently, we attempted to prepare COF-3S using S2 and 1,3,5-tris(4-aminophenyl) benzene (TAPB) as ligands via the solvothermal method. However, the XRD patterns indicated that the obtained products were not microcrystalline materials. Therefore, the solvothermal method is not suitable for the preparation of COF-3X.

We found that ethanethiol, ethylene glycol, and ethylenediamine can undergo substitution reactions with heteroatoms (such as Cl, Br, and I) attached to the benzene ring under alkaline conditions (using cesium carbonate, potassium carbonate, and sodium hydroxide, among others). (Org. Lett. 2020, 22 (21), 8291-8295; J. Am. Chem. Soc. 1953, 75 (7), 1647-1651; J. Am. Chem. Soc. 2000, 122 (51), 12907-12908; Synthetic Commun. 2004, 34 (9), 1723-1727) Therefore, by continuously optimizing conditions such as solvents, alkali dosage, and reaction temperature, we successfully prepared COF3S, COF-3O, and COF-3N. It is worth noting that the Povarov reaction can eliminate imine bonds and enhance the

chemical stability of COFs, thereby minimizing the impact of the two-step reaction on the structure of COFs. Meanwhile, the XRD pattern of COFs also demonstrates that the two-step modification did not cause severe structural damage to the COFs.

Comment 4: In Fig.3b, the experimental data (gray) seems incomplete. The authors are requested to confirm whether the data is complete and to provide data ranging from 2 to 40°.

Response: We appreciate the comment from the reviewer. We have confirmed that the experimental data (in gray) presented in Figure 3b is complete. This data pertains to small-angle X-ray diffraction (XRD), which involves X-ray diffraction measurements conducted within a narrow angular range, typically from 0.1° to 10°. In contrast to traditional XRD, small-angle XRD places greater emphasis on microstructural and nanoscale characteristics of materials, as well as other detailed information. It offers distinct advantages for analyzing specialized materials such as sample surfaces, thin films, nanomaterials, and polymers. This technique can elucidate information regarding pore structures, layered configurations, and variations in crystallinity within materials. In this study, small-angle XRD was employed to correct experimental instrument errors and ascertain structural information about the material-similar to the methodologies illustrated in the figure below.

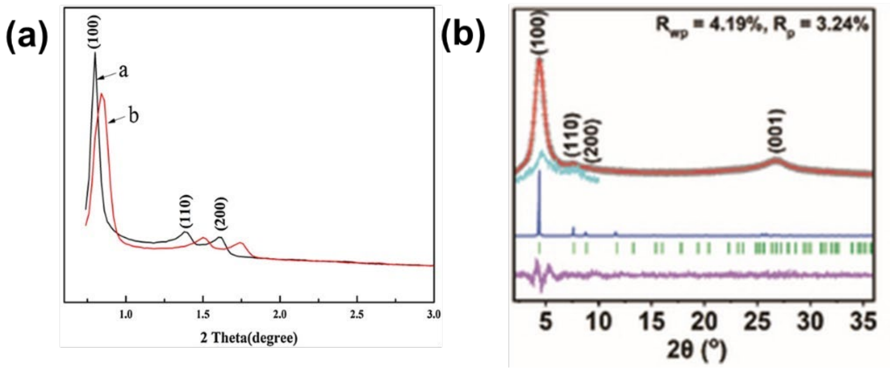

(a) Powder SAXRD patterns of SBA-15 and TBP-SBA-15. (Appl. Surf. Sci. 2017, 402, 53-60.)
(b)

XRD patterns of COF-1 with refinement. Pawley refinement, simulated results, and Bragg positions are in red, blue, and green, respectively. Experimental data (gray cross) display little differences
(purple) with simulation. SAXS data were used for zero-shift correction. (Research 2024, 7.)

Comment 5: Which specific position does the PDOS segments in Figure 6 refer to? In my opinion, it is necessary to insert the schematic diagrams of each fragment in Figures 6g, h, and i.

Response: We appreciate the comment from the reviewer. We acknowledge that it was an oversight on our part not to clearly indicate the fragment locations. In response, we have revised Figures 6g, h, and i to enhance clarity regarding the fragment positions as described in the manuscript for our readers' benefit. The updated figures are presented below.

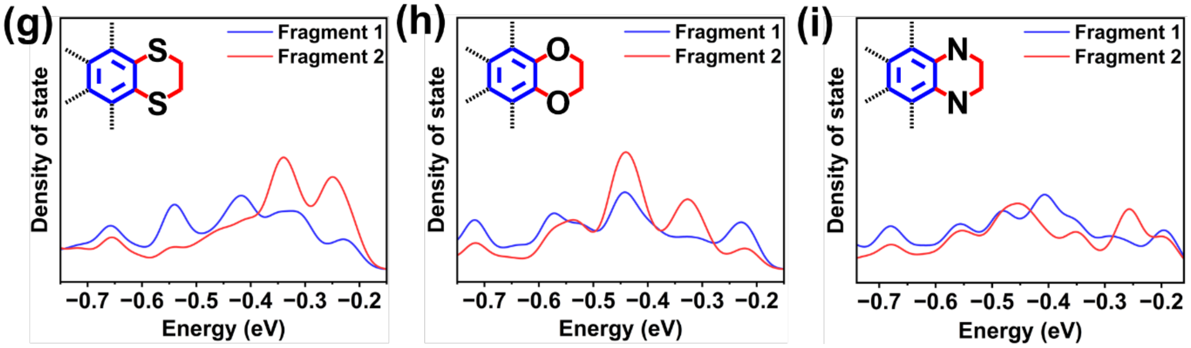

The PDOS curves of COF-3S (g), COF-3O (h), and COF-3N (i).

Reviewer #2:

In this manuscript, Ma and co-workers designed and prepared a series of heteroatom-doped COFs, aiming to investigate the relative importance of conjugation and induction effects in photocatalytic reactivity. The concept stands out among numerous works related to COFs, specifically in terms of the proposal of the induction effect. Even for highly conjugated COF, if it does not possess electronrich regions such as heteroatoms (N, O, or S), the photocatalytic performance seems unlikely to be particularly outstanding. In the previous works focused on the study of COFs as photocatalysts, researchers tend to concentrate solely on the conjugation effect, while ignoring the induction effect. Crucially, both induction and conjugation effects belong to the electronic effects, which determine the electron transfer process within the COF photocatalysts. The manuscript is well-organized, exhibits high novelty, and provides logical results that sufficiently support the conclusions. Meanwhile, the proposed strategies have certain universality, which is expected to draw the broad interests of researchers. Therefore, this manuscript meets the standards required by ACS Central Science, and it is recommended for publication after minor revision.

Special comments:

Response: We are grateful to the reviewer for taking the time to evaluate our work and greatly appreciate their positive comments and support.

Comment 1: Can COF-3S, COF-3O, and COF-3N be directly synthesized? If so, there is no need to prepare COF-2. It is necessary to state in revised form.

Response: We appreciate the comment from the reviewer. We have attempted to prepare COF-3X directly via the solvothermal method. According to the experimental method proposed by Ye et al., we have successfully prepared 2,3,7,8-tetrahydrobenzo[1,2-b:4,5-b']bis([1,4]dithiine)-5,10dicarbaldehyde (S2). (Nat. Commun. 2022, 13 (1), 6116.) Subsequently, we attempted to prepare COF-3S employing S2 and 1,3,5-tris(4-aminophenyl) benzene (TAPB) as ligands via the solvothermal method. However, the results indicated that the obtained product did not possess a crystalline structure. Thus, the synthesis of COF-2 is reasonably necessary. COF-2 is obtained from COF-1 through the Povarov reaction. On the one hand, COF-2 exhibits stronger chemical stability compared to COF-1, ensuring the smooth progress of post-modification. On the other hand, after the Povarov reaction, the two ligands that constitute COF-2 are connected through a quinoline ring, which presents a lower energy barrier compared to imine bonds, making it more conducive to the electron transfer from the donor to the acceptor, reducing the influence of other factors, and thus facilitating the subsequent mechanism investigation. In summary, the design and preparation of COF-2 are of crucial importance throughout the entire process.

Comment 2: The authors mentioned "Subsequently, post-synthetic modification transformed imine bonds to chemically stable chromenoquinoline rings through the Povarov reaction, increasing πconjugation." If the purpose of the Povarov reaction is to increase π conjugation, it seems that it does not make a significant contribution to the exploration of the mechanism. It is suggested that the core purpose of this step be explained in the Introduction.

Response: We greatly appreciate the constructive suggestions from the reviewer. In the manuscript, Povarov reaction serves two purposes: (i) First, the Povarov reaction can increase the degree of π conjugation and enhance the stability of COFs, providing a basis for subsequent secondary modification; (ii) Second, Povarov reaction can eliminate imine bonds, which exhibit directionality and affect the transfer of electrons from the electron donor to the electron acceptor. Eliminating imine bonds allows us to better investigate the distribution of electrons in the acceptor without being influenced by other factors. (Angew. Chem. Int. Edit. 2021, 60 (26), 14236-14250.) Furthermore, in the introduction, we elaborated on the significance of the Povarov reaction to facilitate a better understanding for readers.

Comment 3: In the Introduction, “one of our developed COF-3S delivered a high photocatalytic uranium removal efficiency in contaminated groundwater and tap water.”, there seems to be a mistake in this sentence. It should be seawater and groundwater. Please check it carefully.

Response: We appreciate the comment from the reviewer. We have carefully reviewed this sentence and revised it to: “As a result, one of our developed COFs, COF-3S, delivered a high photocatalytic uranium removal efficiency in contaminated groundwater and seawater.” The modified part has been highlighted in yellow in the manuscript.

Comment 4: The authors used post-modification technique to synthesize COF-3S, COF-3O and COF3N. Is it possible to synthesize these COFs using solvothermal techniques directly?

Response: We appreciate the comment from the reviewer. According to the previous methods, we have attempted to directly synthesize COF-3S using 2,3,7,8-tetrahydrobenzo[1,2-b:4,5b']bis([1,4]dithiine)-5,10-dicarbaldehyde and 1,3,5-tris(4-aminophenyl)benzene as ligands via the solvothermal method. However, after prolonged adjustment of process conditions, the XRD patterns indicated that we still failed to obtain products exhibiting a satisfactory crystal structure. Therefore, the solvothermal method is not suitable for the preparation of COF-3S, COF-3O, and COF-3N.

Comment 5: It is better to describe the conditions for the equilibrium state of conjugation and induction effects in photocatalyst.

Response: We appreciate the comment from the reviewer. The equilibrium state between conjugation and induction effects is crucial for the photocatalytic performance of COFs. In this manuscript, we utilize two indicators to describe the equilibrium state between conjugation and induction effects: (i) the values of the electronic localization function (ELF); (ii) the partial density of states (PDOS) of COFs. Based on these two parameters, we ultimately concluded that the balance between conjugation and induction effects is crucial. To achieve this goal, the two most critical conditions are the adjustment of conjugation and induction effect strengths.

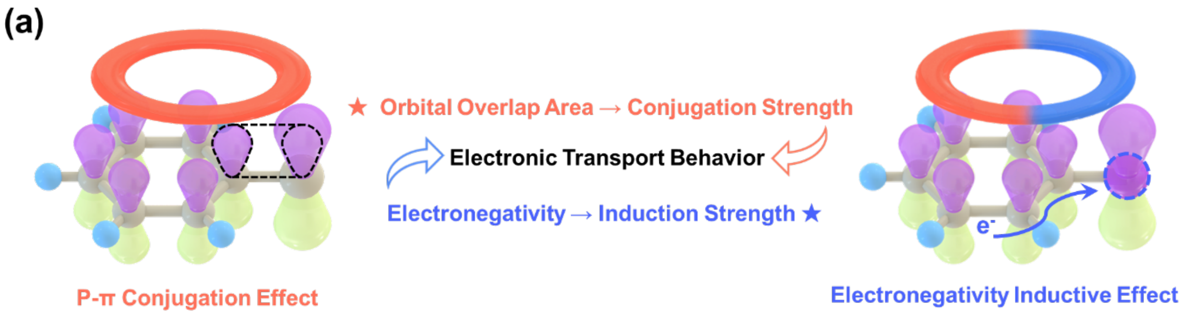

Figure 1a. Schematic illustration of conjugation and induction effects at the molecular level, highlighting the roles of conjugation and induction effects on electron transfer behaviour.

First, as illustrated in Figure 1a, the strength of the conjugation effect is influenced by the overlap area of the P-orbital. Second, the strength of the induction effect is determined by the electronegativity of the heteroatom itself. Consequently, our strategy has been effectively implemented. We would like to express our gratitude once again for your professional suggestions. We have further elaborated on these conditions in the introduction to enhance readers' understanding.

Again, we thank all reviewers for the constructive suggestions, which have made our manuscript much improved.

Sincerely,

Shengqian Ma, PhD
University Distinguished Research Professor
Robert A. Welch Chair in Chemistry

oc-2025-01645w.R2

Name: Peer Review Information for "Systematic Tuning of the Electronic Effects in Covalent Organic Frameworks for Promoting Photocatalysis"

Second Round of Reviewer Comments

Reviewer: 1

Comments to the Author

The authors have revised the manuscript according to my comments, I recommend the publication of the currrent version.

Reviewer: 2

Comments to the Author

Authors have fully addressed my comments.

Author's Response to Peer Review Comments:

Date: November 6, 2025

Senior Editor

ACS Central Science

Dear Editor:

Thank you very much for the provisional acceptance of our manuscript titled "***Systematic Tuning of the Electronic Effects in Covalent Organic Frameworks for Promoting Photocatalysis***" (Manuscript ID: oc-2025-01645w). We greatly appreciate the constructive comments and suggestions from the editorial office, and we have revised our manuscript accordingly, as detailed in the responses below. We hope this revision can be satisfactory for publication in *ACS Central Science*. Thank you for your kind handling of our manuscript.

Editorial office's comments:

Author List: Please include the email address(es) of the corresponding author(s) on the first page of the manuscript.

Response: We have attached the email addresses of the three corresponding authors to the first page of the manuscript.

Synopsis: ACS Central Science requires a brief synopsis. The synopsis should be no more than 200 characters (including spaces) and should reasonably correlate with the Table of Contents (TOC) graphic. The synopsis is intended to explain the importance of the article to a broader readership across the sciences. Please place your synopsis in the manuscript file after the TOC graphic and label as "Synopsis."

Response: We have added the synopsis after the TOC graphic, with the following specific content: A new strategy is reported to optimize the photocatalytic uranyl removal performance of COFs by tuning the relative extent of conjugation and induction effects.

Reviewers' comments:

Reviewer #1:

Recommendation: Publish in ACS Central Science without change.

The authors have revised the manuscript according to my comments, I recommend the publication of the current version.

Response: We are grateful to the reviewer for taking the time to evaluate our work and greatly appreciate their positive comments and support.

Reviewer #2:

Recommendation: Publish in ACS Central Science without change.

Authors have fully addressed my comments.

Response: We are grateful to the reviewer for taking the time to evaluate our work and greatly appreciate their positive comments and support.

Again, we thank all reviewers for the constructive suggestions, which have made our manuscript much improved.

Thank you very much for your favorable consideration of our manuscript.

Sincerely,

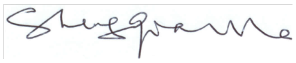

Shengqian Ma, PhD

University Distinguished Research Professor

Robert A. Welch Chair in Chemistry
